# Ancient ubiquitous protein 1 (AUP1) is a prognostic biomarker connected with *TP53* mutation and the inflamed microenvironments in glioma

**DOI:** 10.1186/s12935-023-02912-y

**Published:** 2023-04-07

**Authors:** Pei-Chi Chang, Yu-Chieh Lin, Hui-Ju Yen, Dueng-Yuan Hueng, Shih-Ming Huang, Yao-Feng Li

**Affiliations:** 1 Graduate Institute of Life Sciences, National Defense Medical Center, Taipei, 114 Taiwan, Republic of China; 2 Department of Biochemistry, National Defense Medical Center, Taipei, 114 Taiwan, Republic of China; 3 Department of Pathology, Tri-Service General Hospital, National Defense Medical Center, Taipei, 114 Taiwan, Republic of China; 4 School of Pharmacy & Institute Pharmacy, National Defense Medical Center, Taipei, 114 Taiwan, Republic of China; 5 Department of Neurologic Surgery, Tri-Service General Hospital, National Defense Medical Center, Taipei, 114 Taiwan, Republic of China

**Keywords:** AUP1, Glioblastoma, *TP53*, TCGA, CGGA, GLASS, GSEA, TMB, MSI, CIBERSORT, Single-cell sequencing

## Abstract

**Introduction:**

Glioblastoma (GBM) is the most common and lethal brain tumor. The current treatment is surgical removal combined with radiotherapy and chemotherapy, Temozolomide (TMZ). However, tumors tend to develop TMZ resistance which leads to therapeutic failure. Ancient ubiquitous protein 1 (AUP1) is a protein associated with lipid metabolism, which is widely expressed on the surface of ER and Lipid droplets, involved in the degradation of misfolded proteins through autophagy. It has recently been described as a prognostic marker in renal tumors. Here, we aim to use sophisticated bioinformatics and experimental validation to characterize the AUP1's role in glioma.

**Material and methods:**

We collected the mRNA, proteomics, and Whole-Exon-Sequencing from The Cancer Genome Atlas (TCGA) for bioinformatics analyses. The analyses included the expression difference, Kaplan–Meier-survival, COX-survival, and correlation to the clinical factors (tumor mutation burden, microsatellite instability, and driven mutant genes). Next, we validated the AUP1 protein expression using immunohistochemical staining on the 78 clinical cases and correlated them with P53 and KI67. Then, we applied GSEA analyses to identify the altered signalings and set functional experiments (including Western Blot, qPCR, BrdU, migration, cell-cycle, and RNAseq) on cell lines when supplemented with small interfering RNA targeting the AUP1 gene (siAUP1) for further validation. We integrated the single-cell sequencing and CIBERSORT analyses at the Chinese Glioma Genome Atlas (CGGA) and Glioma Longitudinal AnalySiS (GLASS) dataset to rationale the role of AUP1 in glioma.

**Results:**

Firstly, the AUP1 is a prognostic marker, increased in the tumor component, and correlated with tumor grade in both transcriptomes and protein levels. Secondly, we found higher AUP1 associated with *TP53* status, Tumor mutation burden, and increased proliferation. In the function validation, downregulated AUP1 expression merely impacted the U87MG cells' proliferation instead of altering the lipophagy activity. From the single-cell sequencing and CIBERSORT analyses at CGGA and GLASS data, we understood the AUP1 expression was affected by the tumor proliferation, stromal, and inflammation compositions, particularly the myeloid and T cells. In the longitudinal data, the AUP1 significantly dropped in the recurrent IDH wildtype astrocytoma, which might result from increased AUP1-cold components, including oligodendrocytes, endothelial cells, and pericytes.

**Conclusion:**

According to the literature, AUP1 regulates lipophagy by stabilizing the ubiquitination of lipid droplets. However, we found no direct link between AUP1 suppression and altered autophagy activity in the functional validation. Instead, we noticed AUP1 expression associated with tumor proliferation and inflammatory status, contributed by myeloid cells and T cells. In addition, the *TP53* mutations seem to play an important role here and initiate inflamed microenvironments. At the same time, EGFR amplification and Chromosome 7 gain combined 10 loss are associated with increased tumor growth related to AUP1 levels. This study taught us that AUP1 is a poorer predictive biomarker associated with tumor proliferation and could report inflamed status, potentially impacting the clinical application.

**Supplementary Information:**

The online version contains supplementary material available at 10.1186/s12935-023-02912-y.

## Introductions

The brain tumor is one of the most common cancer and is bound for significant morbidity and mortality worldwide. According to the Central Brain Tumor Registry of the United States (CBTRUS), histology-specific brain tumor statistics reveal that diffuse glioma accounts for 68.5% of primary brain malignancies, with glioblastoma being the most common [1]. Since the revised 4th edition of WHO Classification in 2016 [2] and a series of cIMPACT-NOW updates [3–10], molecular characteristics have played a significant role in predicting prognosis and treatment. These critical molecules include IDH1/2 [11, 12], 1p19q [8], CDKN2A/B homozygous deletions [5], EGFR amplification [9], chromosome 7/10 alterations [9], TERT promoter mutation [9], and MGMT promoter methylation status [13]. In 2021, the new classification book “WHO Classification of Tumors of the Central Nervous System, 5th [14]” was released and adopted the above recommendations. Besides the genes’ status, the tumor methylation features also provided a priceless value in the diagnosis [15].

Despite the advancements in molecular diagnosis, there are still limited treatment options. Currently, the gold standard of patient treatment is gross total resection surgery followed by chemotherapy and radiotherapy, which could maximize survival to approximately 16 months [16–18]. Temozolomide (TMZ) is the standard first-line chemotherapy. However, glioma tends to develop TMZ resistance making the prognosis frustrating. [19]. Hence, it is critical to discover potential therapeutic targets to increase options for chemotherapy. These years, scientists have put a lot of effort into identifying novel targets for brain tumors. A few relevant clinical trials were even completed [20]; however, the results of these trials are suboptimal. Recently, scientists have focused on targets relevant to its metabolic function [21], such as glucose, protein, or lipid metabolism.

In lipid metabolism, the AUP1 plays a critical role to assists lipid droplet degradation through interaction with the Ubiquitin Conjugating Enzyme E2 G2 (UBE2G2) to ligase the ubiquitin, then proceed to the lipophagy pathway [15]. As well as being described on lipid droplets, AUP1 is also widely expressed on ER surfaces, which is involved in misfolded protein degradation by ER-associated degradation [22, 23]. Most recently, it was addressed that AUP1 influences lipid metabolism and accelerates the development of renal clear cell carcinoma by inducing lipid accumulation [24]. In this study, we aimed to characterize the AUP1 in glioma and see if it plays a role similar in renal tumors. Hence, we set numerous sophisticated bioinformatics analyses from the large-scale database and performed the functional modeling using small interfering RNA targeting the *AUP1* gene (siAUP1) to explore its role in glioma.

## Materials and methods

### Data collection for bioinformatics analyses

The examined data collection comprised four segments. First, the primary AUP1 bioinformatics analysis utilized the Cancer Genome Atlas (TCGA) transcriptome dataset (https://portal.gdc.cancer.gov/, accessed April 2022). This dataset encompassed transcriptomes (mRNA) and clinical parameters such as age, gender, tumor grade, histology, overall survival, and vital status. A total of 690 cases were included and shown as (Additional file 1: S1A). However, these cases followed an outdated classification system [25], prompting us to reorganize them into three subgroups based on the 2021 WHO classification [14]: IDH wildtype, IDH mutant astrocytoma, and oligodendroglioma. Additionally, we collected 892 cases of whole-exome sequencing data (.maf) to compute tumor mutation burden (TMB) for further investigation. Second, to validate the transcriptome expression, we used a proteomics dataset from TCGA PDC (https://proteomic.datacommons.cancer.gov/pdc/, accessed April 2022). This dataset contained 110 proteomic samples, including 100 glioblastomas and 10 normal controls (Additional file 1: S1B), with clinical details including patient age, gender, diagnosis, and grade. Thirdly, for the immune cell analysis, we utilized TCGA, Chinese Glioma Genome Atlas (CGGA, http://www.cgga.org.cn/, accessed April 2022), and Glioma Longitudinal AnalySiS (GLASS) data obtained from SYNAPSE (https://www.synapse.org/#!Synapse:syn17038081/wiki/585622, accessed July 2022). Here, we gathered 1012 patient records from CGGA (Additional file 1: S1C) and 176 cases from GLASS (Additional file 1: S1D). In the fourth section, we employed the GLASS dataset, which featured 176 patients: 133 IDH wildtypes (130 cases with paired samples), 31 IDH mutants (28 cases with paired samples), and 12 oligodendrogliomas (10 cases with paired samples) for longitudinal analyses. Lastly, we downloaded two single-cell sequencing datasets from the Gene-Expression Omnibus (GEO) databases: IDH wildtype data (GSE131928, https://www.ncbi.nlm.nih.gov/geo/query/acc.cgi?acc=GSE131928, accessed April 2022) and IDH mutant data (GSE89567, https://www.ncbi.nlm.nih.gov/geo/query/acc.cgi?acc=GSE89567, accessed April 2022).

### Transcriptome, proteomics, and single-cell sequencing data processing

We used R (R version 4.1.0, www.r-project.org) with relevant R packages for the bioinformatics analyses. Firstly, the downloaded gene expression data unit, RPKM (Reads Per Kilobase per Million), was transferred to TPM (Transcripts Per Million). Then, we used limma and ggpubr packages for mRNA quantification, regression, and differentially expressed genes analysis and used the adjusted P-value < 0.05 as a cut-off. Next, we perform the Kaplan–Meier survival and COX-survival analyses to evaluate AUP1's impact on the prognosis. To investigate the effects of the genomic stability, we downloaded the whole exon sequencing data for calculating the TMB using the package "maftools", as well as obtained the Microsatellite Instability (MSI) data from the landmark paper by the team of Sameek Roychowdhury [26]. We analyzed the GSE131928 and GSE89567 datasets for the Single-cell sequencing on the BBrowser platform [27].

### Gene Set Enrichment Analysis (GSEA)

Gene Set Enrichment Analysis (GSEA, https://www.gseamsigdb.org/gsea/index.jsp) [28] is an effective method of computationally analyzing the data in gene expression analyses to obtain the altered signalings by comparing two different groups. Firstly, we used it to investigate the difference in the signalings between high and low-AUP1 expression groups (the cut-off value was set as the median) for TCGA glioma datasets. The geneset used the "hallmark gene-sets v7.5" (accessed in April 2022) as a reference, and the other parameters used default values. In the second part of the GSEA analysis, we performed the functional validation and compared the mRNA sequencing between the U87MG with scrambled siRNA versus U87MG supplemented with siAUP1.

### Twelve-cell state and CIBERSORT analyses

Twelve-cell state and CIBERSORT (https://cibersort.stanford.edu/) analyses are analysis engines developed by Newman et al. at the Alizadeh Lab [29] and Roel G W Verhaak [32]. Through Twelve-cell state deconvolution, we could obtain the critical 12-cell components of the glioma samples [32]. In the CIBERSORT calculations, we could further assess the abundance of types in a mixture of various immune cell populations using the gene expression data. We utilized bulk transcriptome data from the TCGA, CGGA, and GLASS databases as input and determined the proportions of 12 cell components and 22 different immune cells for each case with a significance threshold of p < 0.05, then correlated AUP1 expression.

### Tissue microarray and immunohistochemistry

Firstly, we purchased glioma tissue microarrays (GL1001a) from Biomax, Inc. (https://www.biomax.us/), which contained the tissue sections and clinical information. All human tissue specimens were obtained with the informed consent of donors through US Biomax, Inc. This tissue array included 70 cases of adult glioma (7 for Grade 1; 45 for Grade 2; 10 for Grade 3; 8 for Grade 4) and 8 normal brain tissue (Additional file 1: S1E). The core measured 1.5 mm in diameter with 5 µm tissue thickness. In the immunohistochemistry, the Ventana Benchmark^®^XT immunostainer was used to perform immunohistochemical staining, ensuring consistency. Before staining, samples were heated in a pressure cooker for 30 min at 125 °C for antigen retrieval (0.01 M sodium citrate, pH 6.2), then washed three times in phosphate-buffered saline (PBS) for five minutes each. Next, the slides were uploaded to the autostainer according to the instructions from the manufacturer. The primary antibodies used here include AUP1 (Atlas antibodies #HPA007674), P53 (cell signaling # 2524), and KI67 (Abcam #ab15580). The secondary antibodies used Roche Diagnostics OptiView DAB IHC Detection Kit. Positive and negative controls were used to evaluate the antibodies' binding abilities. Robust staining of the positive control was found, but not that of the negative control.

### Scoring of the immunohistochemistry

As in previous publications [30, 31], stained slides were examined and scored using an automated semi-quantitative system. Firstly, stained slides were digitally scanned, and the tiff files were exported as 10× figures. Then, we used ImageJ Fiji software with macro coding to automate and quantify the entire area using the protocol described in the previous work [31]. Staining areas were rated 0–3, representing negative to strongly positive stains. Tumor-stained area fractions (0–1000‰) were estimated, and summing intensity multiplied by the fraction calculated an Immunoscore (0–3000). Finally, an ANOVA evaluated the significance of immunostaining scores to clinical parameters.

### Human glioma cell lines and lysate preparation

We use the following glioma cell lines for this research, including U87MG, U118MG, LN229, LNZ308, GBM8401, SVGp12, and NHA maintained in DMEM supplemented with 100 units/mL of penicillin, and 100 μg/mL of streptomycin, and 10% fetal bovine serum (FBS). Cultures were kept at 37 °C in an incubator with 5% CO_2_. We determined AUP1 protein expression by Western blotting with lysis buffer containing 100 mM Tris–HCl, 150 mM NaCl, and 1% Triton X-100. Actin, GAPDH, and β-actin acted as internal controls. In addition, normal brain lysate purchased from Biocompare (MBS537208, San Francisco, CA, USA) served as a control.

### Western blot analysis

These cell lines were washed with PBS and then lysed in RIPA buffer (150 mM NaCl, 100 mM Tris–HCl in pH 8.0, 0.1% SDS, and 1% Triton 100). We have separated the protein lysates (20–40 g, according to the concentrations) by 10% SDS-PAGE and analyzed them by immunoblotting with antibodies against polyclonal rabbit anti- AUP1 (Atlas antibodies #HPA007674) and anti-GAPDH (sc-47724, Santa Cruz) antibodies, as well as monoclonal mouse anti-ACTN (sc-17829, Santa. The experiment was repeated, and then we quantified the images using ImageJ (National Institutes of Health, Bethesda, MD, USA).

### RNA extraction and Real-Time PCR

A total RNA extract was prepared using TRIzolTM Reagent (Thermo Fisher Scientific, Waltham, WA, USA), followed by reverse transcription using TetroTM Reverse Transcriptase (Bioline, Taunton, MA, USA). Fast Plus EvaGreen qPCR Master Mix (Biotium, Fremont, CA, USA) was used in qRT-PCR using a StepOneTM Real-Time PCR System (Thermo Fisher Scientific, USA). We used 0.25 m of each primer (PrimerBank) and 2.5 l of cDNA diluent for thermocycling. PCR consisted of two minutes of denaturing at 95 °C, followed by 40 cycles of touch-down PCR (5 s at 95 °C, 30 s annealing at 60 °C). The primers used were as follows: AUP1, 5’-CTTCGTCCTGTTCATCGCCAAC-3’(forward) and 5’-CTTTGTCGCTTCATGTGCTCTGC-3’(reverse); for GAPDH, 5’-GCACCGTCAAGGCTGAGAAC-3’(forward) and 5’-ATGGTGGTGAAGACGCCAGT-3’(reverse).

### Transfection of scrambled siRNA and siAUP1 into Glioma Cell Lines

In this study, we purchased scrambled siRNA and siAUP1 from Dharmacon (Lafayette, product ID, M-012410-01-0005). Through siAUP1, we could downregulate the AUP1 expression for further investigation. The cells were transfected in 6-well plates with 25 nM of siAUP1 or siRNA_scam (Dharmacon, USA) using DharmaFECT 1 Transfection Reagent. After seeding the cells into 6-well plates, each group was incubated overnight at 37 °C with 5 × 10^4^ cells per well. Cells were collected by adding 200 L of 10% FBS DMEM and 100 L of 0.05% trypsin to each well. Finally, a Western blot was performed to detect EMT phenotype (N-cadherin), cell cycle (cyclin D1), autophagy (LC3I, LC3II, P62), mitochondria-biogenesis (mtTFA), the marker-associated reactive oxygen species (Nrf2) expression in the cells following siAUP1 and scrambled siRNA transfection.

### Assessment of the cell cycle

U87MG, LNZ308, and GBM8401 cells were transfected with 25 nM siAUP1 for 72 h and then harvested, fixed with 70% ethanol, washed with PBS/1% FBS, and incubated with 10 mg/mL RNAse A and 50 mg/mL propidium iodide in PBS, plus 1% Tween 20 for 30 min at 37 °C in the dark. Flow cytometric analysis was performed using the FACS Calibur flow cytometer (BD Biosciences NJ, USA). A software program called Cell Quest Pro (BD Biosciences, USA) was used to calculate the fractions in the cell cycle phases.

### Wound healing assays

U87MG, LNZ308, and GBM8401 glioma cell lines were plated in 6-well plates supplemented with mitomycin C at a 5 × 10^4^ cells/well density and transfected with siAUP1 or scrambled siRNA as described above. After incubation for 48 h, a scratch wound was made in the cultures using a 200-µL pipet tip. The cells were washed with PBS, and the scratched area was photographed under a microscope (0 h). One microliter of DMEM supplemented with 10% FBS was added to each well. After incubation for 16 h at 37 °C under 5% CO_2_, the medium was removed, and the scratched area was photographed again.

### Tests to measure the level of reactive oxygen species (ROS) in the cytosol of cells

To detect the production of cytosolic ROS, we plated cells in six-well plates and treated them with metformin to detect ROS production by the cells. After 3 h of treatment with the substance, living cells were stained with 10 mM DCFH-DA (Sigma-Aldrich) for 10 min at 37 °C and then incubated for 3 h. We used flow cytometry to determine the percentage of stained cells.

### Analysis of RNA sequences

The transcriptomes of U87MG cells were collected from six independent experiments, including three control samples supplemented with scrambled siRNA and three other samples containing siAUP1. TRIzol reagent (Invitrogen) was then used to extract total RNA, which was then analyzed at 260 nm, 280 nm, and 230 nm, and the RNA Integrity Number (RIN) values for quality control. Finally, the six samples' RIN values ranged from 9.7 to 10. Then we reverse-transcribed mRNA into 300–500 bp of cDNA and added adapters at both ends using the mRNA Library Kit. RNA sequencing on the NextSeq 550 System was conducted with the adapter fragment attached to a flow cell and amplified with Bridge PCR. A bioinformatics pipeline of the Instrument Center of the National Defense Medical Center provided the expression data shown in counts, RPKM (reads per kilobase of exon per million reads mapped), and TPM (transcripts per million) formats for further investigation.

## Results

### Result 1: AUP1 increased in the tumor component and played a prognostic role in glioma

**Fig. 1:**
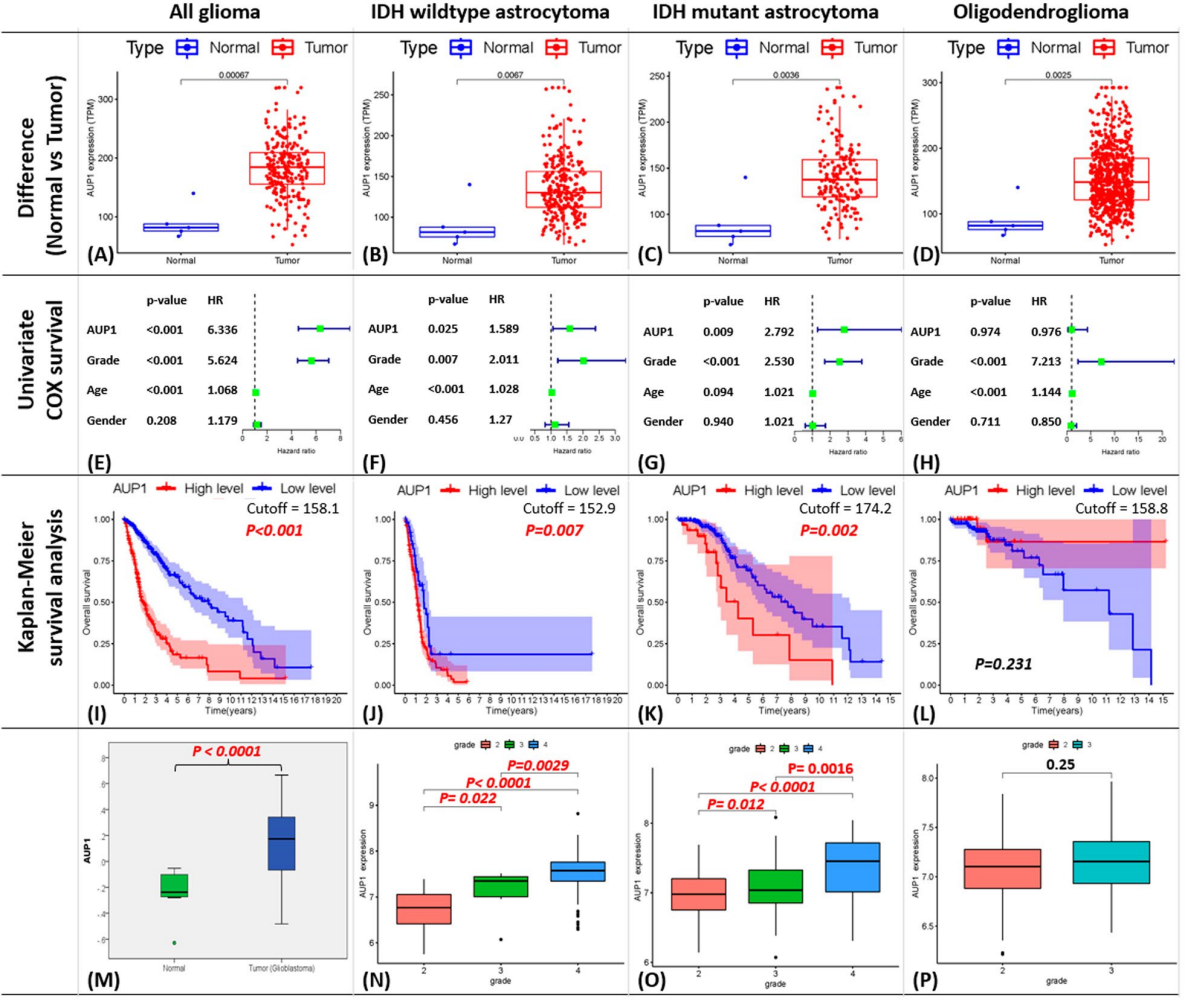
Bioinformatics analyses of AUP1 mRNA and proteomics expression from the TCGA dataset. **A**–**D** Here, we found significant differences in AUP1 expression between normal and tumor in all subgroups. **E**–**H** The results of the univariate-COX survival and **I**, **L** Kaplan–Meier survival analyses support that AUP1 played a poorer prognostic role in astrocytoma groups. **M** Proteomics analysis also showed significantly higher AUP1 expression in tumors. **N**, **O** Additionally, AUP1 was linked substantially with a higher grade in astrocytoma **P** but not with oligodendrogliomas

The bioinformatics analysis of the TCGA dataset revealed that AUP1 significantly increased in tumor components than normal across all subgroups, including pan-glioma, IDH-wildtype, IDH-mutant astrocytoma, and Oligodendroglioma (Fig. 1A–D). In the COX-survival analysis, increased AUP1 significantly correlated with a higher hazard ratio in the IDH-wildtype and IDH-mutant astrocytoma (Fig. 1E–G) but not in the Oligodendroglioma (Fig. 1H). Kaplan–Meier survival analysis demonstrated a poorer prognosis with high AUP1 in pan-glioma, IDH-wildtype, and IDH-mutant astrocytoma (Fig. 1L, K) but not in oligodendroglioma. To verify whether the functional unit, protein, displayed a similar pattern, we also examined TCGA proteomic data for AUP1. As the transcriptomes showed, the AUP1 protein expression was significantly higher in the tumor components than in the normal component (Fig. 1M). When compared with clinical factors, increased AUP1 was significantly related to higher grading in both astrocytoma groups (Fig. 1N, O) but not in oligodendrogliomas (Fig. 1P).

### Result 2: AUP1 correlated with tumor grade, P53 status, and proliferation index (KI67)

**Fig. 2:**
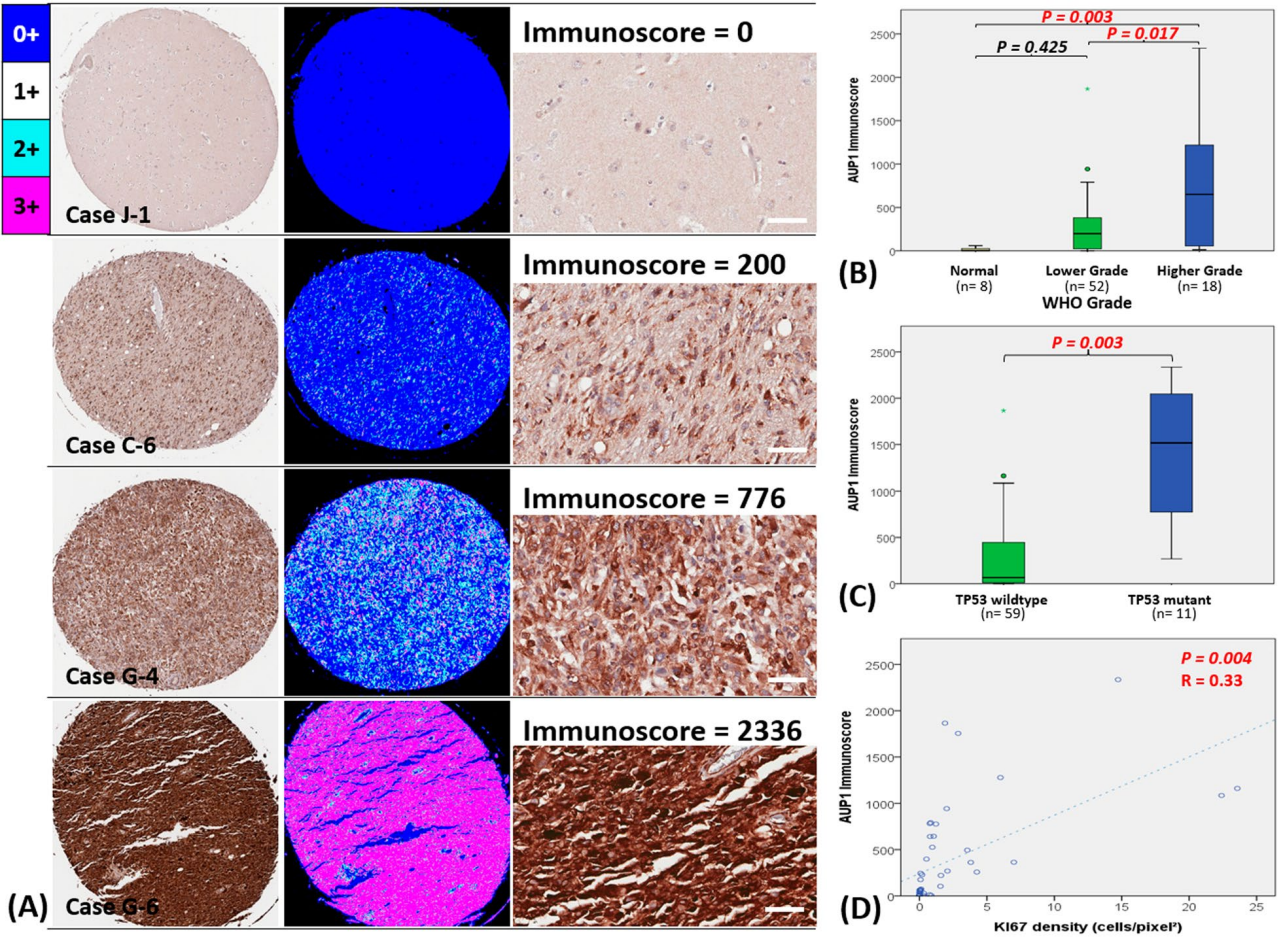
Quantify the AUP1 immunohistochemistry and the correlation with the prognostic markers. **A** Representative cases through ImageJ analysis defined the different intensities from (0 to 3) with corresponding stained-area fractions (0–1000‰) for Immunoscore calculation. **B** The Immunoscore of AUP1 staining positively correlates with tumor grading. **C** In addition, its higher expression is also relevant to the P53 mutation status and **D** increased KI67 labeling index

We further used immunohistochemistry to validate the AUP1 protein expression at the tissue level and analyzed 78 clinical cases. All images were analyzed through ImageJ software into different staining intensities ranging from 0 to 3 + , representing negative to strongly positive stains (Fig. 2A). The stained-area fractions (0–1000‰) of each intensity were also estimated, and then summing intensity multiplied by the fraction calculated an Immunoscore (0–3000, Additional file 2: S2). When correlating the Immunoscore with the clinical prognostic markers, such as age, gender, tumor grade, P53 status, and proliferation index (KI67), the results showed that higher AUP1's Immunoscore is associated with higher tumor grades (Fig. 2B). In addition, we also noticed a significant association between elevated AUP1 expression with the status of mutant P53 (Fig. 2C) and the KI67 labeling index (Fig. 2D).

### Result 3: AUP1 correlates to *TP53* mutation, EGFR amplification, chromosome 7 gain/10 loss, and TMB, but reversely to MSI

**Fig. 3:**
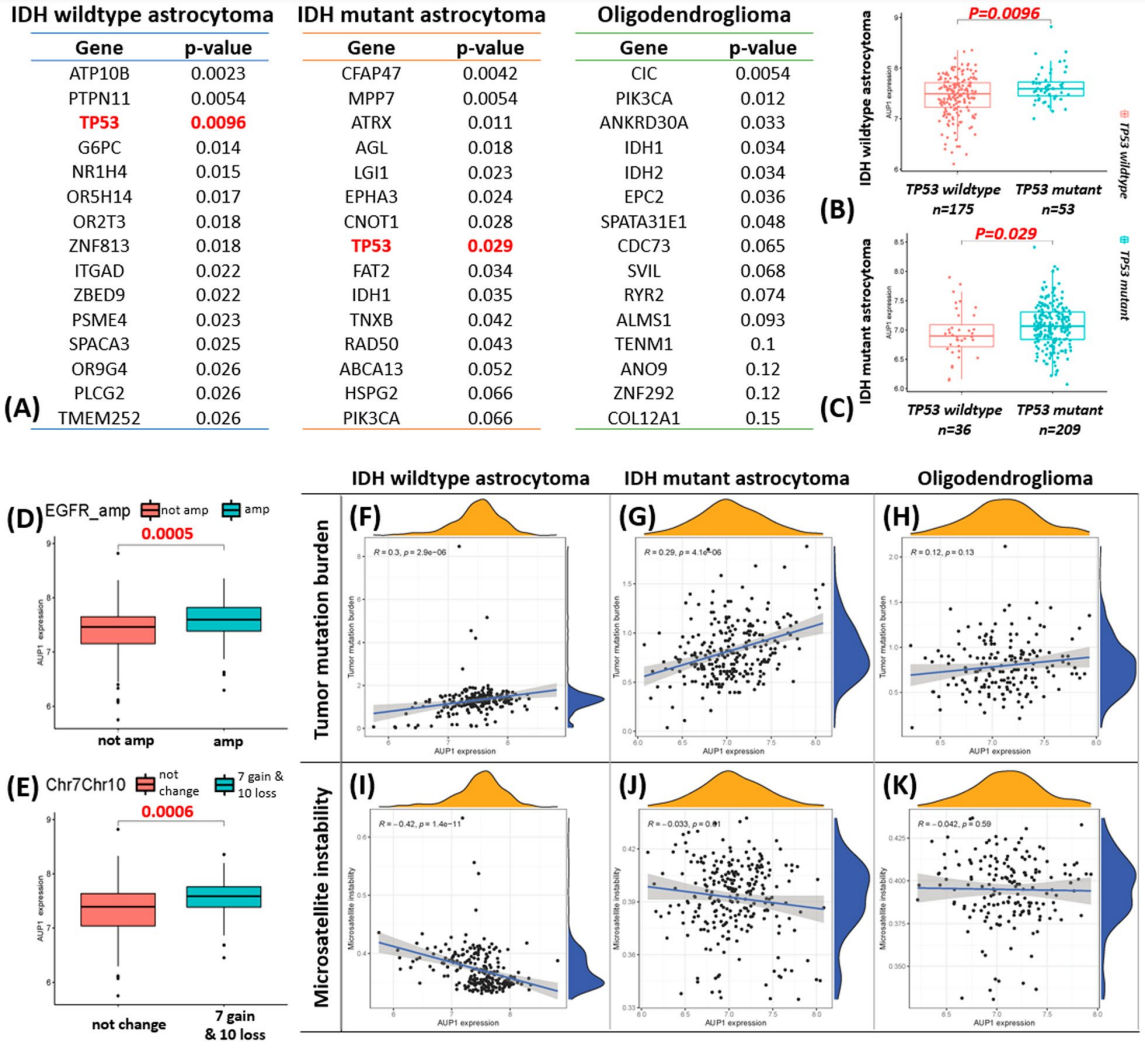
AUP1 expression is associated with gene mutations, chromosome abnormality, and genomic stability. **A** We identified potential driving genes that might impact AUP1 in each group, and there is a strong correlation between *TP53* in astrocytoma groups. **B**, **C** The boxplots revealed the association of AUP1 with *TP53* status in IDH wildtype and mutant astrocytoma groups. **D** Higher AUP1 also correlated with EGFR amplification and **E** chromosome 7 gain and 10 loss. **F**, **G** For genomic stability, the increased AUP1 positively correlated to TMB in astrocytoma (H) but not oligodendroglioma. **I**, **J** Conversely, the AUP1 was reversely associated with MSI in astrocytoma **K** but not oligodendroglioma

To confirm the association between AUP1 and *TP53*, we looked at the transcriptome, whole-exon-sequencing, and chromosome structure abnormalities from the TCGA dataset. Firstly, from screening the AUP1 expression with each gene's mutant status (14,733 genes evaluated, Additional file 3), we got lists of genes in each group that correlated with AUP1 expression (Fig. 3A). Interestingly, on the lists of the IDH wildtype and IDH mutant astrocytoma groups, *TP53* is the only intersected gene, and the boxplot showed as (Fig. 3B, C). This Identification matched the immunohistochemistry findings that the higher AUP1 expression was associated with *TP53* mutation. Secondly, besides the gene mutation, the extrachromosomal DNA (such as EGFR amplification, Fig. 3D) and chromosome structure (such as chromosome 7 gain with 10 loss, Fig. 3E) are associated with higher AUP1 in IDH wildtype astrocytoma. Finally, we looked at the impacts of genomic stability, including the TMB or MSI. The results showed that AUP1 positively correlated to TMB in IDH-wildtype and IDH-mutant astrocytoma (Fig. 3F–G) but not Oligodendroglioma (Fig. 3H). On the contrary, the AUP1 expression was reversely associated with MSI in astrocytoma groups (Fig. 3I, J) but not Oligodendroglioma (Fig. 3K).

### Result 4: The higher AUP1 expression is associated with cell proliferation signaling

**Fig. 4:**
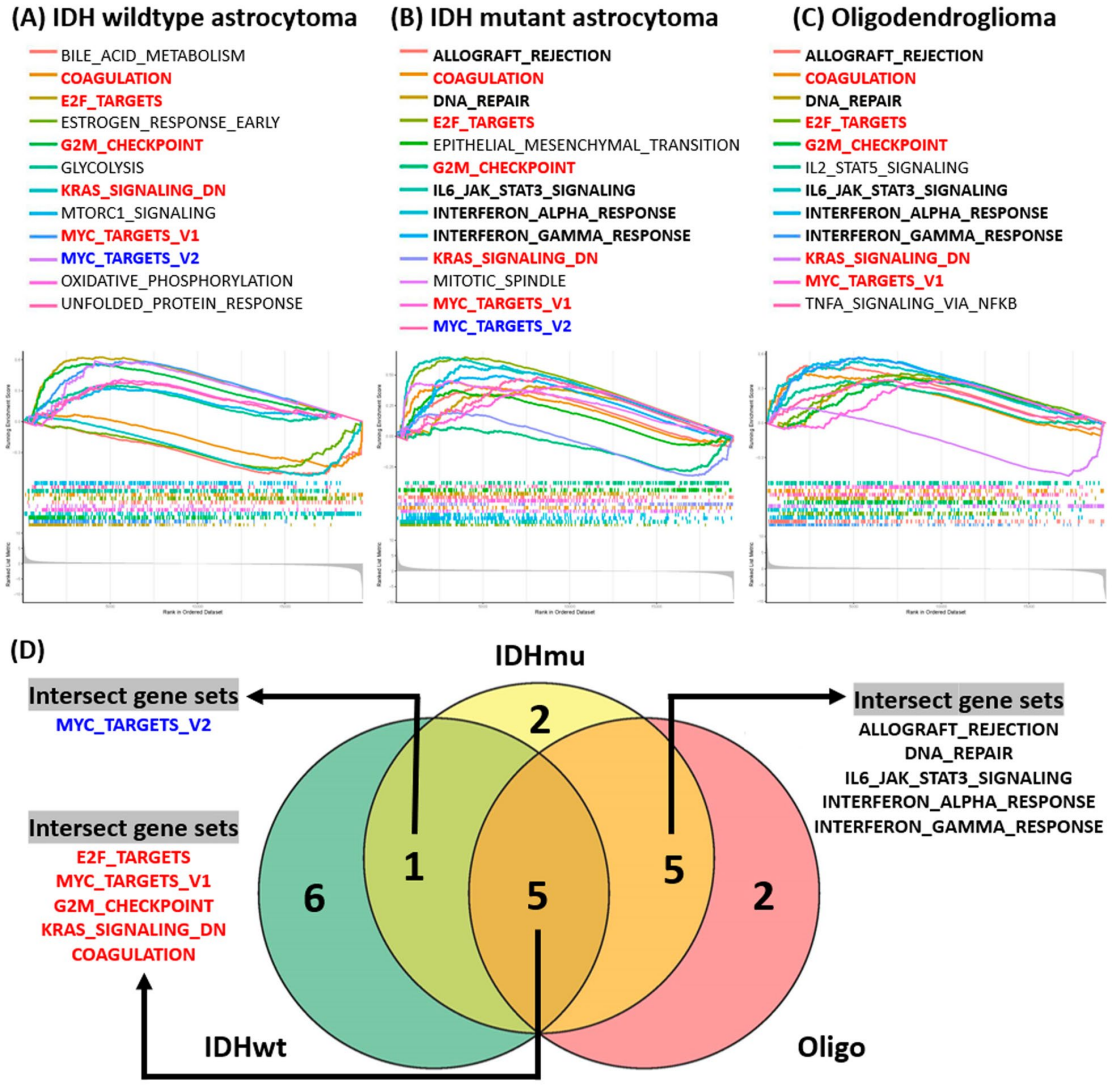
Gene Set Enrichment Analysis (GSEA). **A**–**C** From the GSEA analyses, we identified the altered signalings relevant to the higher AUP1 expression in each subgroup. **D** The intersected gene sets between each subgroup were listed. The crossed gene sets among all subgroups were E2F_TARGETS, MYC_TARGETS_V1, G2M_CHECKPOINT, KRAS_SIGNALING, and COAGULATION

To understand the altered signaling when AUP1 expression increased, we use Gene Set Enrichment Analysis (GSEA) to characterize the TCGA datasets further. The results revealed the altered signalings relevant to the higher AUP1 expression in each subgroup (Fig. 4A–C). Surprisingly, the lists show many intersected gene sets among these subgroups, including E2F_TARGETS, MYC_TARGETS_V1, G2M_CHECKPOINT, KRAS_SIGNALING, and COAGULATION. The first 3 gene sets are associated with cell proliferation, which supported the findings that AUP1 correlated with KI67 expression. In the astrocytomas groups, there is another crossed gene set, MYC_TARGETS_V2 (Fig. 4D), which was also relevant to cell growth. For the IDH mutant astrocytoma and oligodendroglioma, there are the other 5 intersected gene sets, including ALLOGRAFT_REJECTION, DNA_REPAIR, IL6_JAK_STAT3_SIGNALING, INTERFERON_ALPHA_RESPONSE, and INTERFERON_GAMMA_RESPONSE, which were mainly associated with increased inflammation (Fig. 4D).

### Result 5: In the function validation, down-regulation of AUP1 affected U87MG proliferation, not the others

**Fig. 5:**
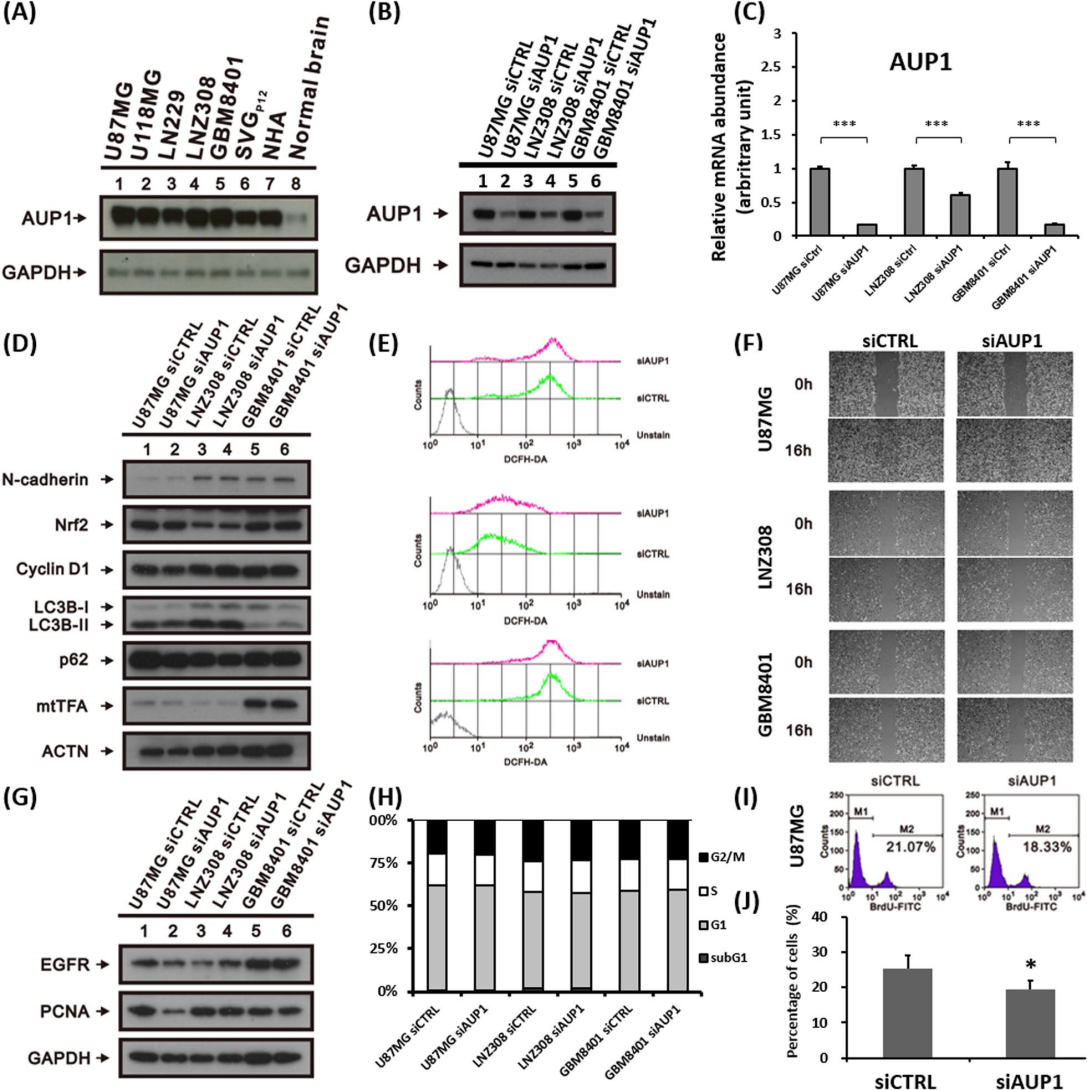
The function validation by down-regulated the AUP1 expression. **A** The protein expression of AUP1 revealed a marked increase compared to the normal control. **B**, **C** When knowing down the AUP1 gene, its mRNA expression efficiently downregulated in both proteins and transcriptome levels. **D** Several markers relevant to invasiveness, ROS, and autophagy were examined, including N-cadherin, Nrf2, mtFTA, p62, LC3B1, and LC3BII. **E** We also observed the cytosolic ROS and **F** migration ability after being supplemented with siAUP1; however, the results are inconspicuous. **G** Then, we checked the proliferation markers, including EGFR and PCNA, and the results showed a marked decrease in the U87MG cells. **H** But the changes in the cell cycle in U87MG, LNZ308, and GBM8401 were not obvious. **I**, **J** In the BrdU analyses of U87MG, the proportion of the cells in the S phase revealed significantly decreased when supplemented with siAUP1. Bars, mean ± SEM; *p < 0.05, **p < 0.01, ***p < 0.005

To further validate the AUP1's functional role, we use siAUP1 to downregulate the AUP1's expression to investigate associated phenotypes. Firstly, we confirmed that AUP1 expression at several tumor cell lines, including the U87MG, U118MG, LN229, LNZ308, and GBM8401. The results showed that the tumor cells revealed higher AUP1 expression than the normal brain lysates (Fig. 5A), similar to the previous bioinformatics analyses. Next, we applied the siAUP1 in the selected cell lines, including U87MG, LNZ308, and GBM8401, and witnessed the marked decreased AUP1 expression in both proteins (Fig. 5B) and transcriptome levels (Fig. 5C). From the literature [15], the AUP1 was known to play an essential role in the autophagy pathway, so we checked the relevant markers, and here we found that p62 did show a decreased trend, but the LC3BI and LC3BII remained unchanged when down-regulated AUP1 (Fig. 5D). Next, we investigated several markers relevant to the cancer hallmarks, such as N-cadherin for Epithelial-mesenchymal transition (EMT), mtFTA for mitochondria biogenesis, and Nrf2 for reactive oxygen species (ROS); however, no apparent alteration was identified (Fig. 5D). To further check the ROS alteration when supplemented with siAUP1, we did not identify the changes in the cytosolic ROS in all three cell lines (Fig. 5E). In the migration assay, we did not find a slower migration when knocked down AUP1 (Fig. 5F), which further confirmed the unchanged EMT capability. In the previous GSEA analyses, we discovered many changes in signaling associated with cell proliferation. Hence, we check a few proliferation markers, including the EGFR and Proliferating cell nuclear antigen (PCNA), and the cell-cycle analysis. The results showed that one of the examined cell lines, U87MG, marked decreased proliferation markers at the EGFR and PCNA (Fig. 5G) when supplemented with siAUP1. Although the changes in the cell cycle analyses are not noticeable (Fig. 5H), the BrdU staining significantly further supported that the downregulated AUP1 deteriorated proliferation in the U87MG (Fig. 5I-J).

### Result 6: Functional modeling supports the effect of higher AUP1 on proliferation and ***TP53*** signaling

**Fig. 6:**
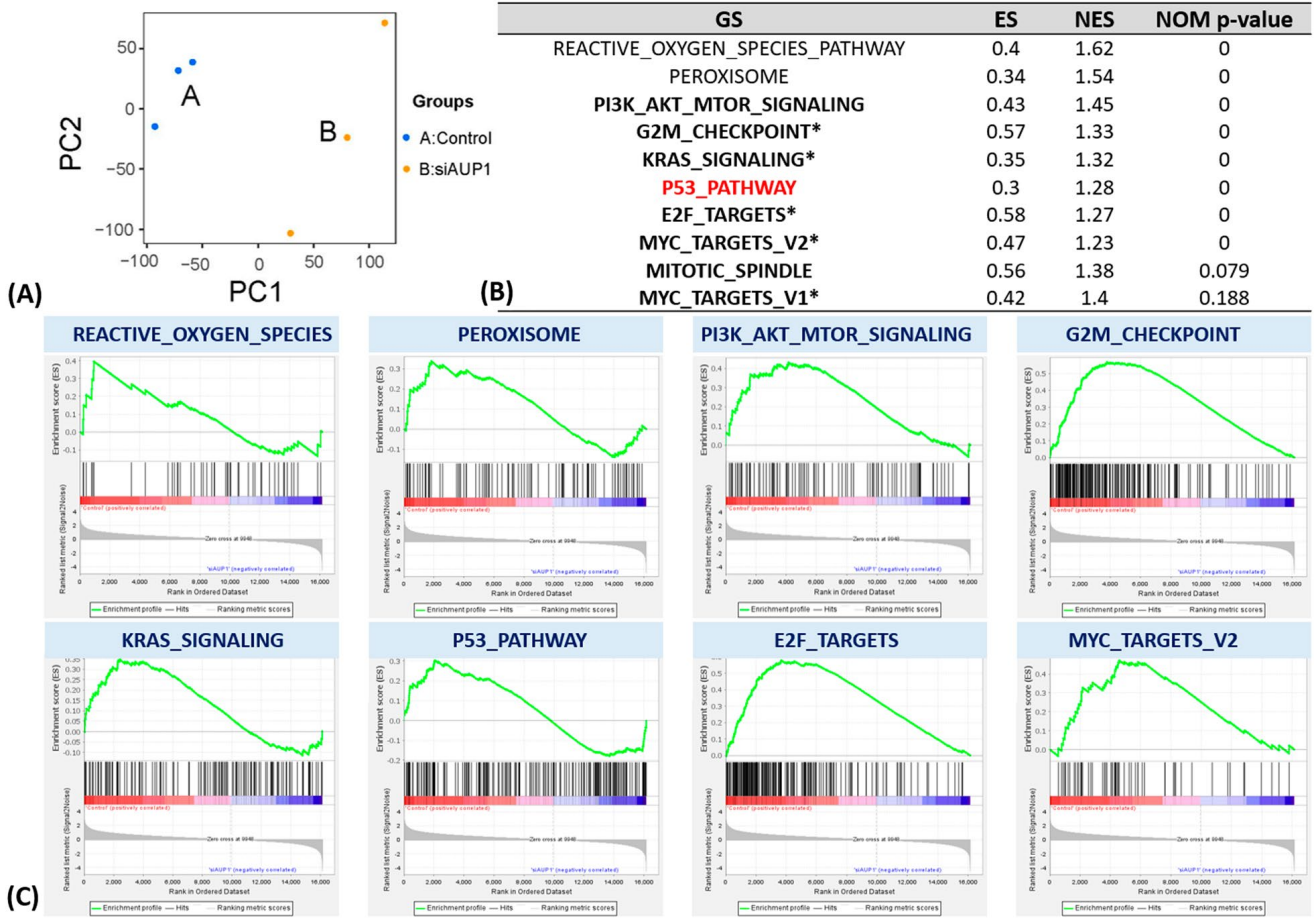
The GSEA analyses the mRNA sequencing between U87MG with scrambled siRNA and U87MG with siAUP1. **A** The PCA plot showed the transcriptomes of each group were different. **B**, **C** The results of the GSEA showed that there were eight significantly increased signalings in the control group, including the increase of REACTIVE_OXYGEN_SPECIES, PEROXISOME, PI3K_AKT_MTOR_SIGNALING, G2M_CHECKPOINT, KRAS_SIGNALING, P53_PATHWAY, E2F_TARGETS, and MYC_TARGETS_V2. Most of them are relative to the proliferation ability

To confirm the results of the GSEA and previous functional modeling that the AUP1 was relevant to the proliferation signaling. We performed the total mRNA sequencing and compared the control group (relatively higher AUP1) with the U87MG supplemented with siAUP1 (relatively lower AUP1). The data showed in Additional file 4: S4). Firstly, from the PCA plot, the control samples formed a cluster away from the siAUP1 treatment group (Fig. 6A) without intermixing, indicating that the transcriptome features are unique. The results of GSEA analyses compared the control group versus U87MG with siAUP showed a significantly increased of REACTIVE_OXYGEN_SPECIES, PEROXISOME, PI3K_AKT_MTOR_SIGNALING, G2M_CHECKPOINT, KRAS_SIGNALING, P53_PATHWAY, E2F_TARGETS, and MYC_TARGETS_V2 (Fig. 6B, C). Most are relative to the proliferation ability and similar to the previous GSEA analyses from the TCGA datasets. Interestingly, the increased *TP53* signaling also existed in the higher AUP1 group (see Additional file 5).

### Result 7: In addition to the tumor cells, the inflammatory cells also contributed to AUP1 expression

**Fig. 7:**
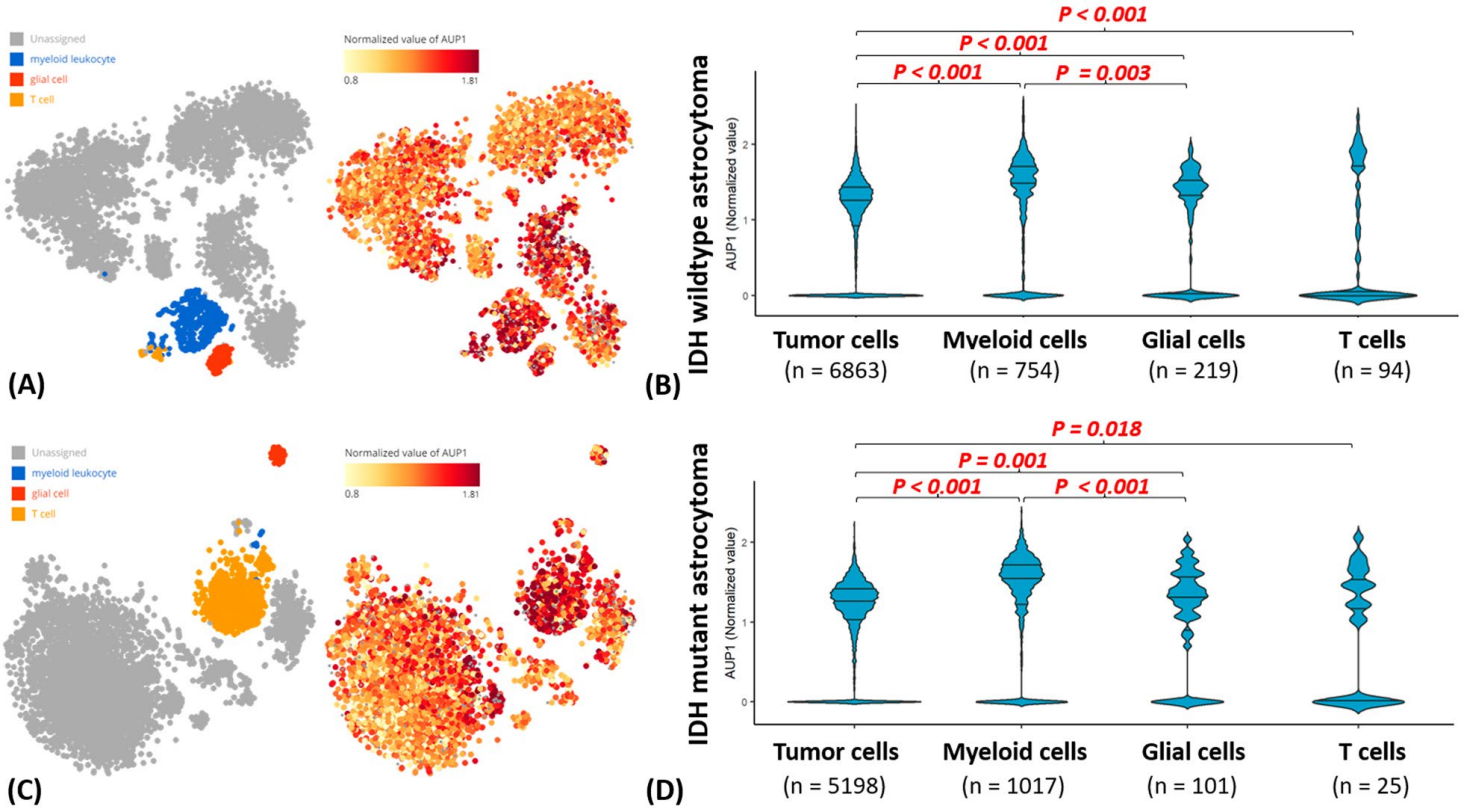
The AUP1 expression in the single-cell sequencing analyses. **A**, **B** In the heatmap of AUP1 expression and cell type annotations, we noticed AUP1 highly correlated with myeloid T cells, partly tumor components in IDH-wildtype astrocytoma. The difference between myeloid to tumor cells or reactive glia reached statistical significance. **C**, **D** The IDH mutant astrocytoma showed a similar trend that AUP1 expression was highly associated with myeloid and T cells, also called the statistic significance

The earlier bioinformatics characterization and functional validation showed that AUP1 correlated with tumor grade and proliferation function. However, some of the cell lines didn't show apparent phenotype change when supplemented with siAUP1, and we also noticed a few inflammatory singling in the GSEA analyses. Hence, we are considering whether any other factor linked to the AUP1 could also be associated with the prognosis. Most of the previous analyses were based on the bulk RNA sequencing of the tumor tissue; however, the tissue intermixed with numerous cell heterogeneity, such as the tumor, inflammatory cells, endothelial cells, etc. To investigate whether tumor heterogeneity impacts the AUP1 expression, we analyzed two single-cell sequencing databases, GSE131928 and GSE89567, representing IDH wildtype and IDH mutant astrocytoma, respectively. The heatmap of the AUP1 expression showed that the expression highly matched with myeloid and T cell lineages in the IDH wildtype astrocytoma (Fig. 7A). The difference between myeloid to tumor cells or reactive glial cells reached statistical significance p < 0.001 and p = 0.003, individually (Fig. 7B). The results of the IDH mutant astrocytoma group showed a similar trend that the AUP1 also correlated with myeloid and T cells (Fig. 7C). The difference between myeloid to tumor cells and myeloid to reactive glia also reached statistical significance, revealing p < 0.001 (Fig. 7D). Then, we further characterized the relationship between the AUP1 with the PI3K-AKT-MTOR pathway, proliferation markers, and autophagy signaling for further investigation. From the results, we noticed that tumor cells highly expressed AUP1 significantly correlated with proliferation marker (MCM2, MCM6), PI3K-AKT-MTOR pathway (Akt1, TSC1, mTOR, Foxo1), and autophagy signaling (Atg3, Atg4B, Atg4D, Atg7, Atg9A, Atg16L1) in IDH wildtype tumor cells (Table 1). Similarly, in the context of the IDH mutant astrocytoma, the tumor with higher AUP1 was significantly correlated with PI3K-AKT-MTOR pathway (EGFR, PDGFRA, TSC2, MTOR), and autophagy signaling (ATG4B) (Table 2). In the IDH mutant group, we also noticed significantly increased autophagy activities (ATG2A, ATG3, ATG4B) in the myeloid cells (Table 2).

**Table 1:** IDH wildtype astrocytoma

Factors	Biomarker	AUP1	AUP1	AUP1	AUP1
	Cell types	Tumor (n=6836)	Myeloid (n=754)	Glia (n=219)	T_cells (n=94)
Age	Correlation	0.008	0.038	− 0.11	− 0.129
	Sig.	0.49	0.301	0.104	0.215
Gender	Correlation	− 0.017	− 0.063	− 0.042	0.033
	Sig.	0.164	0.082	0.54	0.75
MCM2	Correlation	0.043**	0.068	− 0.05	− 0.119
	Sig.	**0**	0.061	0.46	0.254
MCM6	Correlation	0.038**	− 0.064	0.062	0.025
	Sig.	**0.002**	0.077	0.358	0.81
AKT1	Correlation	0.029*	0.054	0.021	0.106
	Sig.	**0.017**	0.141	0.755	0.311
TSC1	Correlation	0.029*	− 0.051	.156*	− 0.043
	Sig.	**0.016**	0.162	**0.021**	0.682
MTOR	Correlation	0.039**	0.041	− 0.024	− 0.171
	Sig.	**0.001**	0.266	0.723	0.099
FOXO1	Correlation	0.024*	0.033	0.069	− 0.102
	Sig.	**0.046**	0.359	0.313	0.328
ATG3	Correlation	0.046**	0.063	− 0.072	− 0.078
	Sig.	**0**	0.082	0.29	0.452
ATG4B	Correlation	0.038**	0.019	− 0.013	− 0.089
	Sig.	**0.002**	0.596	0.843	0.395
ATG4D	Correlation	0.032**	0.026	0.011	− 0.117
	Sig.	**0.008**	0.477	0.877	0.26
ATG7	Correlation	0.021	0.023	− 0.058	− 0.118
	Sig.	0.085	0.531	0.393	0.256
ATG9A	Correlation	0.040**	0.003	0.128	0.055
	Sig.	**0.001**	0.929	0.058	0.598
ATG16L1	Correlation	0.048**	0.023	0.026	0.204*
	Sig.	**0**	0.535	0.701	**0.048**
SQSTM1	Correlation	0.016	0.004	0.194**	− 0.112
	Sig.	0.194	0.906	**0.004**	0.283

**Table 2:** IDH Mutant astrocytoma

Factor	Biomarker_AUP1	AUP1	AUP1	AUP1	AUP1
	Cell types	Tumor (n=5198)	Myeloid (n=1017)	Glia (n=101)	T_cells (n=25)
Grade	Correlation	0.071**	0.075*	0.165	− 0.483*
	Sig.	**0**	**0.017**	0.099	**0.014**
Age	Correlation	0.048**	0.023	− 0.199*	0.384
	Sig.	**0.001**	0.465	**0.046**	0.058
Gender	Correlation	− 0.048**	− 0.013	− 0.061	− 0.031
	Sig.	**0.001**	0.687	0.546	0.884
MCM2	Correlation	0.011	− 0.054	− 0.065	0.314
	Sig.	0.438	0.083	0.518	0.127
EGFR	Correlation	− 0.085**	0.025	0.084	− 0.239
	Sig.	**0**	0.422	0.405	0.25
PIK3R1	Correlation	− 0.026	0.015	− 0.208*	− 0.061
	Sig.	0.065	0.625	**0.037**	0.773
AKT2	Correlation	0.024	0.086**	− 0.041	0.014
	Sig.	0.088	**0.006**	0.686	0.949
AKT3	Correlation	− 0.016	− 0.038	0.285**	0.22
	Sig.	0.245	0.229	**0.004**	0.29
TSC2	Correlation	0.029*	0.071*	− 0.175	− 0.277
	Sig.	**0.034**	**0.024**	0.081	0.181
MTOR	Correlation	0.028*	0.025	− 0.047	0.066
	Sig.	**0.041**	0.42	0.639	0.753
ATG2A	Correlation	− 0.024	0.093**	0.115	− 0.036
	Sig.	0.078	**0.003**	0.253	0.865
ATG3	Correlation	− 0.008	0.101**	0.14	0.168
	Sig.	0.549	**0.001**	0.161	0.422
ATG4B	Correlation	0.034*	0.062*	− 0.053	0.279
	Sig.	**0.013**	**0.047**	0.6	0.177
ATG5	Correlation	0.026	− 0.016	− 0.208*	− 0.073
	Sig.	0.059	0.61	**0.037**	0.728
ATG7	Correlation	0.02	0.034	0.326**	− 0.08
	Sig.	0.14	0.275	**0.001**	0.702
ATG9A	Correlation	0.019	0.046	− 0.208*	0.021
	Sig.	0.181	0.142	**0.037**	0.922
ATG13	Correlation	0.017	0.079*	− 0.165	0.07
	Sig.	0.229	**0.012**	0.099	0.74
ATG16L1	Correlation	0.026	− 0.031	0.121	− 0.148
	Sig.	0.057	0.329	0.228	0.479
SQSTM1	Correlation	− 0.003	− 0.022	0	0.328
	Sig.	0.816	0.492	1	0.109

### Result 8: The AUP1 is confirmed to be associated with tumor proliferative stem cells, stroma, and immune microenvironments using computation prediction

**Fig. 8:**
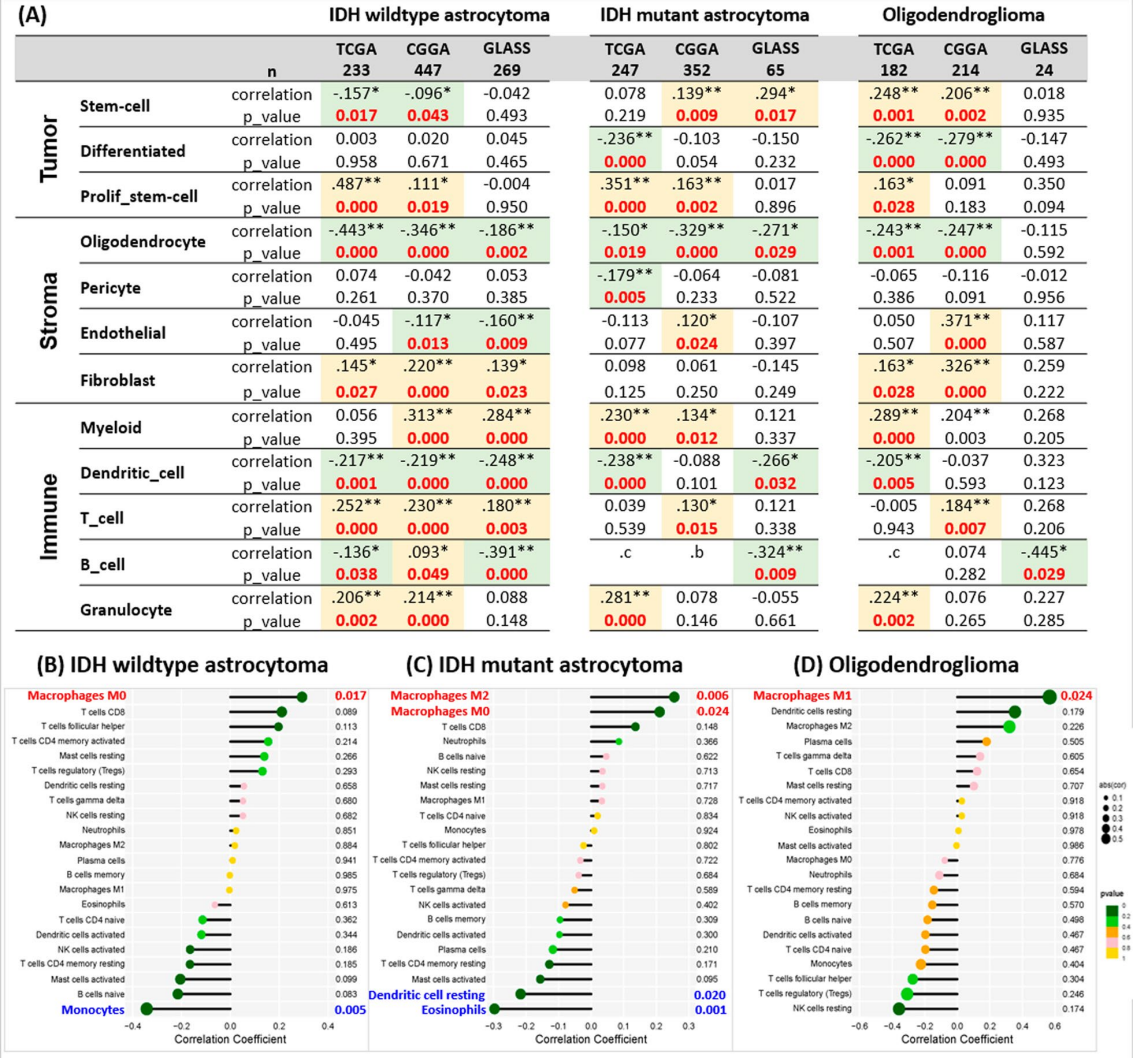
The correlation with AUP1 expression with 12-cell state and 22 different immune cells through deconvoluted bulk transcriptome from TCGA, CGGA, and GLASS. **A** The AUP1 is significantly associated with tumor proliferation, factors of stromal activities, and numerous immune compositions. **B** The CIBERSORT analyses showed that AUP1 had related considerably to increased M0 macrophage in IDH wildtype; **C** M0 and M2 macrophage in IDH mutant astrocytoma; **D** M1 in the oligodendroglioma. The yellow background indicates the significant correlation, while the green represents the significant negative correlation

To further dissect the relation between AUP1, myeloid, T cells, and the other cell components, we deconvoluted the bulk transcriptomes into 12 critical cellular states established by Prof. Roel G W Verhaak's team [32] and the 22 immune cells by Alizadeh Lab [29] to correlate them with AUP1 expression. In the tumor components, we found that AUP1 correlates positively with proliferative stem cells in all three subtypes among different databases (Fig. 8A), which is compatible with previous experimental validation. On the contrary, there are reversed trends in differentiated tumor cells. In addition, the AUP1 showed a negative association with IDH wildtype astrocytoma but positive to the IDH mutant glioma (Fig. 8A). In the stroma cell components, the AUP1 was positively associated with fibroblast but negative to oligodendrocyte amounts. In the endothelium, the AUP1 positively correlated to the IDH mutant glioma but negatively to the IDH wildtype astrocytoma. In the immune cell components, we noticed the myeloid and T cell amounts are positively correlated with AUP1 expression, which is similar to the findings of the single-cell sequencing analyses. In addition, we noticed several changes that have not been seen in the single cells studies, including the increase of granular cells and drop of dendritic cells. For the B cells, most of the datasets showed a significantly decreased, except the IDH wildtype astrocytoma of the CGGA data, which only showed borderline significance (*p* = *0.049*) and weak correlation (r = 0.093).

To further investigate the association of the immune components with the AUP1, we used CIBERSORT to characterize further. From the results, the AUP1 was significantly associated with increased M0 macrophage and depletion of monocytes in IDH wildtype astrocytoma (Fig. 8B). In the IDH mutant astrocytoma, AUP1 revealed a significant association not only with M0 macrophage but also with M2 macrophage (Fig. 8C). For Oligodendroglioma, AUP1 is significantly associated with M1 macrophage (Fig. 8D). From the above results, we have confirmed the findings from the single-cell sequencing that the expression of AUP1 was relevant to the myeloid lineages, particularly for the M0, M1, and M2 macrophages. However, there is a slight difference among different subtypes, and no significant association was found with T cells from the TCGA dataset.

### Result 9: The AUP1 expression dropped during recurrence in the IDH wildtype glioma

**Fig. 9:**
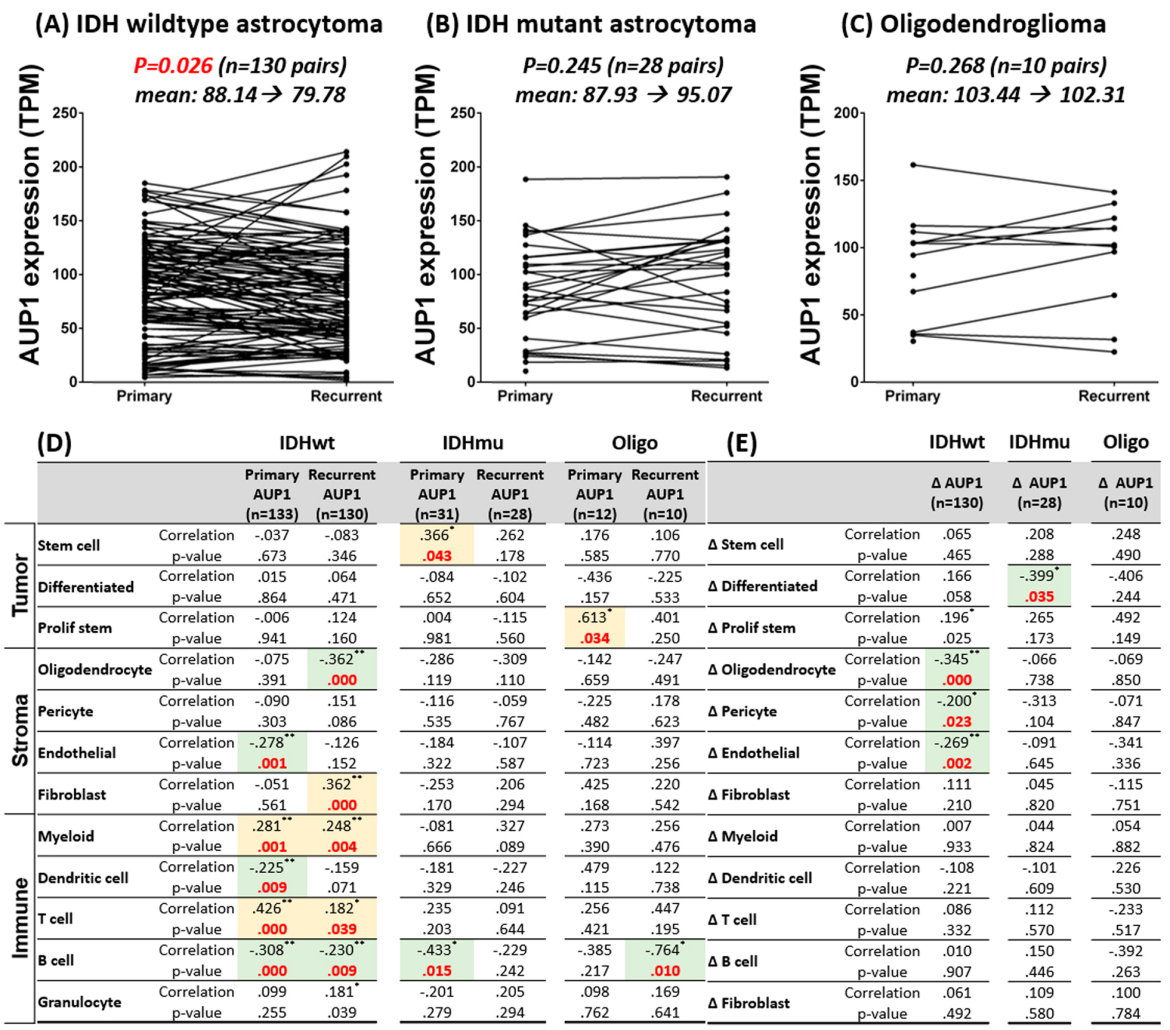
Analysis of AUP1 expression in the Glioma Longitudinal AnalySiS (GLASS) dataset. **A** AUP1 showed a significant decrease when recurrence occurred in the IDH wildtype **B**, **C** but not in IDH mutant and oligodendroglioma. **D** The correlation between primary and recurrent AUP1 with 12 cellular states in glioma. **E** AUP1 differences are reversely related to oligodendrocytes, endothelial cells, and pericytes in IDH wildtype astrocytoma. The yellow background indicates the significant correlation, while the green represents the significant negative correlation

Next, we investigate the dynamic changes in AUP1 between primary and recurrent tumors using the Glioma Longitudinal AnalySiS (GLASS) dataset. The database contained 168 paired studies, including 130 IDH wildtypes, 28 IDH mutants, and 10 oligodendrogliomas. From the analysis, AUP1 showed a significant decrease when recurrence occurred in the IDH wildtype (Fig. 9A). In contrast, there is no significant difference in AUP1 expression in the other two groups (Fig. 9B, C). To further dissected the explanation behind this, we investigate the AUP1 expression with 12 critical cellular states [32]. In the IDH wildtype astrocytoma, it was found that AUP1 significantly correlated with higher myeloid and T cells but reversed to B cells, regardless of whether the cancer was primary or recurrent. During tumor recurrence, AUP1 levels increased with fibroblasts (Fig. 9D) but inverted to oligodendrocytes (Fig. 9D). To discover possible explanations for the decrease of AUP1, we correlated the difference between primary and recurrent AUP1 (∆_AUP1) with the difference of each cell state. We hypothesized that the critical factor causing the decline in AUP1 should be closely related to the difference in AUP1. Results showed that AUP1 differences are reversely related to the difference of the oligodendrocytes, endothelium, and pericytes (Fig. 9E), which are AUP1 cold populations.

## Discussion

In recent years, brain tumor diagnosis has improved considerably, but treatment options remain limited. Hence, identifying the potential therapeutic target is one of the unmet medical needs. Here, we focused on one of the potential targets, AUP1, which is critical in lipid metabolism. The current understanding of AUP1 is limited to assisting the degradation of lipid droplets through interaction with UBE2G2 to stabilize the ubiquitin and proceed to the lipophagy process [15]. In cancer research, the AUP1 has even been discovered to influence lipid metabolism and accelerate the development of renal clear cell carcinoma by inducing lipid accumulation recently [24]. In this project, we wonder if AUP1 also plays a similar role in glioma.

Through bioinformatics analyzing the TCGA genomic and proteomic datasets, we noticed the AUP1 did increase in the tumor component, relevance to the tumor grading, and associated with overall survival. Due to the previous publication addressing the AUP1's role in lipophagy [15], we first validated the autophagy-related markers (p62, LC3BI, and LC3BII) in the Western Blot. However, we could only identify the decrease of ubiquitin, p62, when downregulated AUP1. There were no apparent changes in LC3BI and LC3BII, representing the quantifies of autophagosomes and autophagy activities. Hence, the alteration of the autophagy signaling can not explain why higher AUP1 links with poorer prognosis here. Therefore, the AUP1 might play some roles that haven't been identified to influence survival.

From the intersected gene sets of the GSEA results from the TCGA, we saw the altered signaling mainly relevant to cell proliferation, including E2F_TARGETS, MYC_TARGETS_V1, and G2M_CHECKPOINT. Therefore, we set the functional modeling to validate if the proliferation function will drop when downregulating the AUP1 expression. Our results showed that one of the cell lines, U87MG, fit our hypothesis, which showed an apparent decrease in EGFR and PCNA, and BrdU results when supplemented with siAUP1. To further confirmed the role of AUP1 in the proliferation. We sequenced mRNA from control and U87MG with siAUP1 for further GSEA analysis. The results of the functional modeling revealed a significant increase in REACTIVE_OXYGEN_SPECIES, PEROXISOME, PI3K_AKT_MTOR_SIGNALING, G2M_CHECKPOINT, KRAS_SIGNALING, P53_PATHWAY, E2F_TARGETS, and MYC_TARGETS_V2 in the high AUP1 group. Most altered singlings were associated with cell proliferation and closely matched the initial GSEA analyses of the TCGA data. More Interestingly, the increased *TP53* signaling also existed in the control group (higher AUP1 group), which further supports our findings that the AUP1 expression seems to link with *TP53* status. However, one issue still questions us the other two cell lines, LNZ308 and GBM8401, remained unchanged in most of the tested experiments, including EMT, mitochondria biogenesis, ROS, etc. Hence, we assumed that the high AUP1 expression might still link with the other factors that affect the prognosis apart from the above-tested ones.

At first, the results of the bioinformatics analyses on the AUP1 were established based on the bulk mRNA sequencing from the TCGA database. Hence, the AUP1 expression might also be affected by the cellular heterogeneity of the examined tissue. Hence, the AUP1 expression might also be affected by cellular heterogeneity. To answer this question, we used single-cell sequencing datasets to dissect the role of cellular heterogeneity at AUP1. The heatmap of the single-cell sequencing analyses showed that the tumor cells revealed variable expression, and only part of them showed high AUP1 expression. In addition, we noticed that the inflammatory cells, including myeloid and T cells, surprisingly contributed to the AUP1 expression in both IDH wildtype and IDH mutant astrocytoma (Fig. 6A, C). To further dissect the impact of the cellular heterogeneity at the AUP1, we use the powerful bioinformatics tools 12-cell state prediction [32] and CIBERSORT [29] to deconvolute the mRNA into the different cell states and 22 immune compositions. The results showed that AUP1 positively correlates with proliferative stem cells in all three subtypes (Fig. 8A) in TCGA, CGGA, and GLASS databases, similar to the previous GSEA results and functional validation. In the immune cell composition, we identified that the myeloid cells, T cells, and granulocytes were positively associated with AUP1 expression. Next, we used CIBERSORT to dissect the myeloid lineages further and compare AUP1. TCGA data analyses showed that AUP1 expression was significantly associated with increased M0 macrophage in IDH wildtype, M0 and M2 macrophage in IDH mutant, and M1 macrophage in oligodendroglioma. In addition, we even identified a significant drop in the monocytes in the IDH wildtype group, which might result from their transformation into macrophages and cause the depletion of monocytes [33]. The reversed relation between the monocytes and macrophage could lead to masking subtle changes when observed together, such as using the 12-cell states analyses instead of separating 22 immune cells using CIBERSORT. For the T cells, there is no considerable relevance between AUP1 and any T cell in the TCGA dataset when breaking down the T cells into different subtypes. In addition, the dendritic cells and B cells revealed a negative association in all large-scale databases, including TCGA, CGGA, and GLASS, which were not identified in the single-cell sequencing. However, the B cells' results showed decreased trends with slight contrast in the IDH wildtype astrocytoma of the CGGA, which only revealed borderline significance (*p* = *0.049*) and weak correlation (r = 0.093). In the previous publication [32], the accuracy of B cell prediction was suboptimal compared to the other cell types, and more studies need to confirm further. For the stromal cells, the AUP1 is associated with increased amounts of fibroblast, which could be relevant to the endothelial proliferation that requires fibroblasts, which tend to occur in higher-grade tumors.

So far, we have understood the amount of AUP1 linked to the myeloid cells and partly tumor cells. But we still don't know which factor induced the myeloid cells to gather. While investigating the potential factor that affected the AUP1 upregulation, we noticed that lists of gene mutations might play a role, particularly the *TP53*, which showed in both IDH wildtype and IDH mutant groups. From the literature, the scientist noticed that the *TP53* mutant exhibited increased CCL2 and TNFA secretion by nuclear factor kappa B (NF-κB), resulting in increased microglia and monocyte-derived immune cell infiltration [34]. This piece of research supported two points in current studies. Firstly, it directly explained the macrophage might be derived from microglia and monocytes through NF-kB signaling. This association has been recently reported as well [33]. Secondly, it also demonstrated the potential reason the monocytes were depleted, which was also identified in our study (Fig. 8A).

To further understand the longitudinal changes of AUP1 in glioma patients, we analyzed the primary and recurrent tumors from the Glioma Longitudinal AnalySiS (GLASS) dataset. The results showed that the AUP1 showed a trend of slightly decreased in the recurrent IDH wildtype astrocytoma (Fig. 9A). To clarify the potential explanation behind this, we deconvoluted the bulk sequencing data of the GLASS datasets into the principal 12-cell states and performed the statistical analysis. From the data, we noticed the AUP1 expression positively correlated with most of the inflammatory cells, including the Myeloid, Dendritic cell, and T cell, but reversely to the B cells. The findings match the results of the single cells sequencing data and CIBERSORT analyses from the TCGA, that the myeloid and T cells are the primary resources of AUP1, apart from the tumor. However, these could only tell us the distribution of AUP1 among the different cell types. In order to realize the truth behind the decrease of the AUP1 in the recurrent tumor, we compared the difference of AUP1 with the contrast of the other cell state. The genuine factor should be significantly correlated with the changes of the AUP1 (∆_AUP1). The results showed that the AUP1 difference was reversed to the changes of oligodendrocytes, endothelial cells, and pericytes. These cellular populations were the AUP1 cold population from the first part of the GLASS analysis (Fig. 9D). In other words, when these components increased, the proportion of the AUP1 expressed population decreased relatively, resulting in lower AUP1 at the recurrent tumor.

## Conclusion

The current project is the first study to characterize the association between the role of AUP1 in glioma. From the literature, AUP1 plays a vital role in lipid metabolism by stabilizing the ubiquitination of the lipid droplet for lipophagy. However, our data did not find a direct linkage of altered autophagy activity when suppressed AUP1. Instead, we noticed AUP1 expression associated with tumor proliferation, the number of myeloid cells, dendritic cells, T cells, B cells, granulocytes, and some stromal cells. Hence, the AUP1 could reflect the fibroblast formation and inflamed status, which tends to occur in higher-grade tumors, such as grade 4 glioblastoma. In addition, the driven mutation, *TP53,* might play a critical role in initiating these inflamed microenvironments. At the same time, EGFR amplification and Chromosome 7 gain combined 10 loss are associated with increased tumor growth related to AUP1 levels. Through this study, we learned that AUP1 is a poorer predictive biomarker associated with tumor proliferation and could report inflamed microenvironments in the tissue, potentially a clinical application.

## Supplementary Information

**Additional file 1.** Summary of the case number in each bioinformatic and immunohistochemistry analysis.

**Additional file 2.** Immunohistochemistry quantification for AUP1, IDH, P53, & KI67.

**Additional file 3.** Mutation matrix of 892 GBMLGG cases.

**Additional file 4.** Transcriptomes of U87MG with or without siAUP1.

**Additional file 5.** Original data of Western-Blot for Fig. 5.

## Data Availability

Data used in this study can be accessed via the TCGA GDC portal (https://portal.gdc.cancer.gov/), CGGA (http://www.cgga.org.cn/), GLASS (https://www.synapse.org/#!Synapse:syn17038081/wiki/585622) and the Gene-Expression Omnibus (GEO) databases (GSE89567, https://www.ncbi.nlm.nih.gov/geo/query/acc.cgi?acc=GSE89567). NGS sequencing data for the glioma cell line investigation are available at GSE227721 (https://www.ncbi.nlm.nih.gov/geo/query/acc.cgi?&acc=GSE227721).

## Abbreviations

AUP1: Ancient ubiquitous protein
CBTRUS: Central brain tumor registry of the United States
CGGA: Chinese glioma genome atlas
GEO: Gene-expression omnibus
GLASS: Glioma longitudinal analysis
GSEA: Gene set enrichment analysis
LDs: Lipid droplets
MSI: Microsatellite instability
RPKM: Reads per kilobase of exon per million
siAUP1: Small interfering RNA targeting the AUP1 gene
siRNA: Small interfering RNA
TCGA: The Cancer Genome Atlas
TMB: Tumor mutation burden
TPM: Transcripts per million
UBE2G2: Ubiquitin Conjugating Enzyme E2 G2
