# Supplementary material for: Ancient ubiquitous protein 1 (AUP1) is a prognostic biomarker connected with TP53 mutation and the inflamed microenvironments in glioma
Source: Cancer Cell Int. 2023 Apr 7;23:62. doi: 10.1186/s12935-023-02912-y (PMC10080956; doi:10.1186/s12935-023-02912-y)
Supplement: Supplementary file 1 — Additional file 1. Summary of the case number in each bioinformatic and immunohistochemistry analysis. [file 12935_2023_2912_MOESM1_ESM.docx]

**Supplementary 1.** Summary of the case number in each bioinformatic and immunohistochemistry analysis

**A.** The primary mRNA analyses for this project utilized independent samples downloaded from the TCGA (for **Figure 1**, **Figure 3**, **Figure 4**, and **Figure 8**). Raw data can be accessed at <https://portal.gdc.cancer.gov/>.

| 2007 adult glioma classification | N |  |  | 2021 adult glioma classification | N | |
| --- | --- | --- | --- | --- | --- | --- |
| Grade 2 oligodendroglioma | 115 |  | **Oligo** | Grade 2 oligodendroglioma | 183 | 101 |
| Grade 2 oligoastroctyoma | 77 |  |  | Grade 3 oligodendroglioma |  | 82 |
| Grade 2 astrocytoma | 65 |  | **IDHmu** | Grade 2 IDH mutant astrocytoma | 247 | 124 |
| Grade 3 oligodendroglioma | 82 |  |  | Grade 3 IDH mutant astrocytoma |  | 101 |
| Grade 3 oligoastroctyoma | 55 |  |  | Grade 4 IDH mutant astrocytoma |  | 22 |
| Grade 3 astrocytoma | 130 |  | **IDHwt** | Grade 2 IDH wildtype astrocytoma | 232 | 14 |
| Grade 4 glioblastoma | 166 |  |  | Grade 3 IDH wildtype astrocytoma |  | 10 |
|  |  |  |  | Grade 4 IDH wildtype astrocytoma |  | 208 |
| Total | **690** |  |  | **Total** |  | **662*** |

*There were 28 cases cannot be further classified into the 2021 system due to insufficient molecular information.

**B.** The AUP1 protein validation was performed using independent samples downloaded from TCGA-PDC (for **Figure 1M**). Raw data can be accessed at <https://pdc.cancer.gov/pdc/>.

| Histology diagnosis | N |
| --- | --- |
| Grade 4 glioblastoma | 100 |
| Normal brain tissue | 10 |
| Total | 110 |

**C.** The immune cellular composition analyses utilized 12 cell states and CIBERSORT, based on an extra mRNA dataset from CGGA consisting of independent samples (for **Figure 8**). Raw data can be accessed at <http://www.cgga.org.cn/>.

| 2007 adult glioma classification | N |  |  | 2021 adult glioma classification | N | |
| --- | --- | --- | --- | --- | --- | --- |
| Grade 2 oligodendroglioma | 112 |  | **Oligo** | Grade 2 oligodendroglioma | 213 | 106 |
| Grade 2 oligoastroctyoma | 9 |  |  | Grade 3 oligodendroglioma |  | 107 |
| Grade 2 astrocytoma | 175 |  | **IDHmu** | Grade 2 IDH mutant astrocytoma | 351 | 130 |
| Grade 3 oligodendroglioma | 92 |  |  | Grade 3 IDH mutant astrocytoma |  | 141 |
| Grade 3 oligoastroctyoma | 21 |  |  | Grade 4 IDH mutant astrocytoma |  | 80 |
| Grade 3 astrocytoma | 215 |  | **IDHwt** | Grade 2 IDH wildtype astrocytoma | 444 | 53 |
| Grade 4 glioblastoma | 388 |  |  | Grade 3 IDH wildtype astrocytoma |  | 98 |
|  |  |  |  | Grade 4 IDH wildtype astrocytoma |  | 293 |
| Total | **1012** |  |  | **Total** |  | **1008** |

*There were 4 cases cannot be further classified into the 2021 system due to insufficient molecular information.

**D.** Longitudinal AUP1 mRNA analyses were conducted on paired samples from GLASS (for **Figure 8** and **Figure 9**). Raw data can be accessed at <https://www.synapse.org/#!Synapse:syn17038081/wiki/585622>.

| 2007 adult glioma classification | N |  |  | 2021 adult glioma classification | N | |
| --- | --- | --- | --- | --- | --- | --- |
| Grade 2 oligodendroglioma | 12 |  | **Oligo** | Grade 2 oligodendroglioma | 12  **(10 cases with paired sample)** | 9 |
| Grade 2 oligoastrocytoma | 6 |  |  | Grade 3 oligodendroglioma |  | 3 |
| Grade 2 astrocytoma | 9 |  | **IDHmu** | Grade 2 IDH mutant astrocytoma | 31  **(28 cases with paired sample)** | 19 |
| Grade 3 oligodendroglioma | 3 |  |  | Grade 3 IDH mutant astrocytoma |  | 6 |
| Grade 3 oligoastrocytoma | 4 |  |  | Grade 4 IDH mutant astrocytoma |  | 6 |
| Grade 3 astrocytoma | 9 |  | **IDHwt** | Grade 2 IDH wildtype astrocytoma | 133  **(130 cases with paired sample)** | 0 |
| Grade 4 glioblastoma | 133 |  |  | Grade 3 IDH wildtype astrocytoma |  | 8 |
|  |  |  |  | Grade 4 IDH wildtype astrocytoma |  | 125 |
| Total | **176** |  |  |  |  | **176** |

**E.** In addition, AUP1 protein validation was conducted on Formalin-Fixed Paraffin-Embedded tissue sections using immunohistochemistry from Biomax Tissue microarray (for **Figure 2**). Relevant information can be accessed at <https://www.tissuearray.com/Tissue_Arrays>.

|  | N | Histology diagnosis | N |
| --- | --- | --- | --- |
| Grade 1 | 7 | Grade 1 astrocytoma | 7 |
| Grade 2 | 45 | Grade 2 oligodendroglioma | 2 |
|  |  | Grade 2 oligoastrocytoma | 1 |
|  |  | Grade 2 astrocytoma | 42 |
| Grade 3 | 10 | Grade 3 oligodendroglioma | 1 |
|  |  | Grade 3 oligoastrocytoma | 1 |
|  |  | Grade 3 astrocytoma | 8 |
| Grade 4 | 8 | Grade 4 glioblastoma | 8 |
| Normal | 8 | Normal brain tissue | 8 |
| Total | 78 |  | 78 |
