# Supplementary material for: Ancient ubiquitous protein 1 (AUP1) is a prognostic biomarker connected with TP53 mutation and the inflamed microenvironments in glioma
Source: Cancer Cell Int. 2023 Apr 7;23:62. doi: 10.1186/s12935-023-02912-y (PMC10080956; doi:10.1186/s12935-023-02912-y)

Supplementary 4_for **Figure 5(A)**

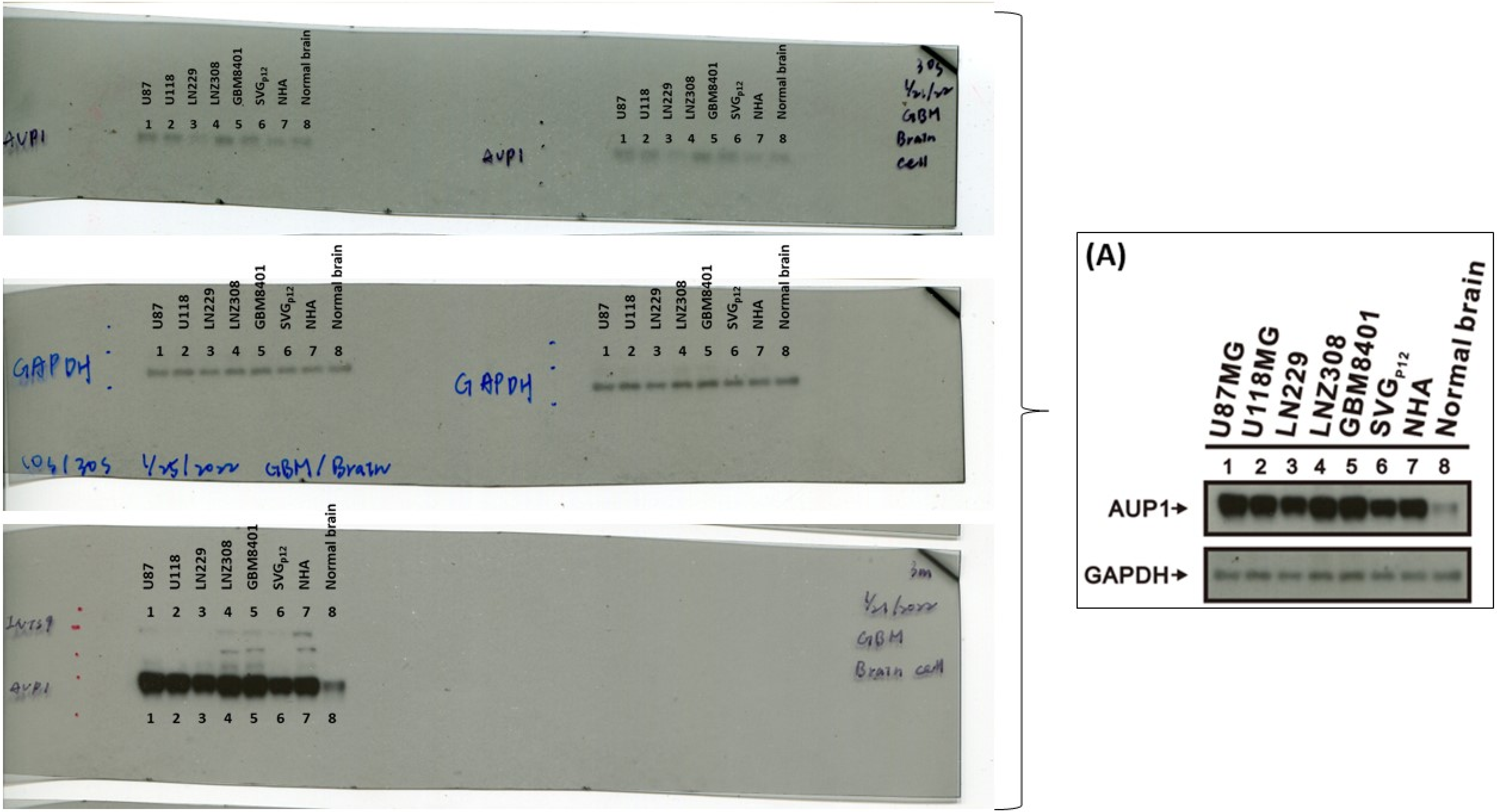

Supplementary 4 _for **Figure 5(B)**

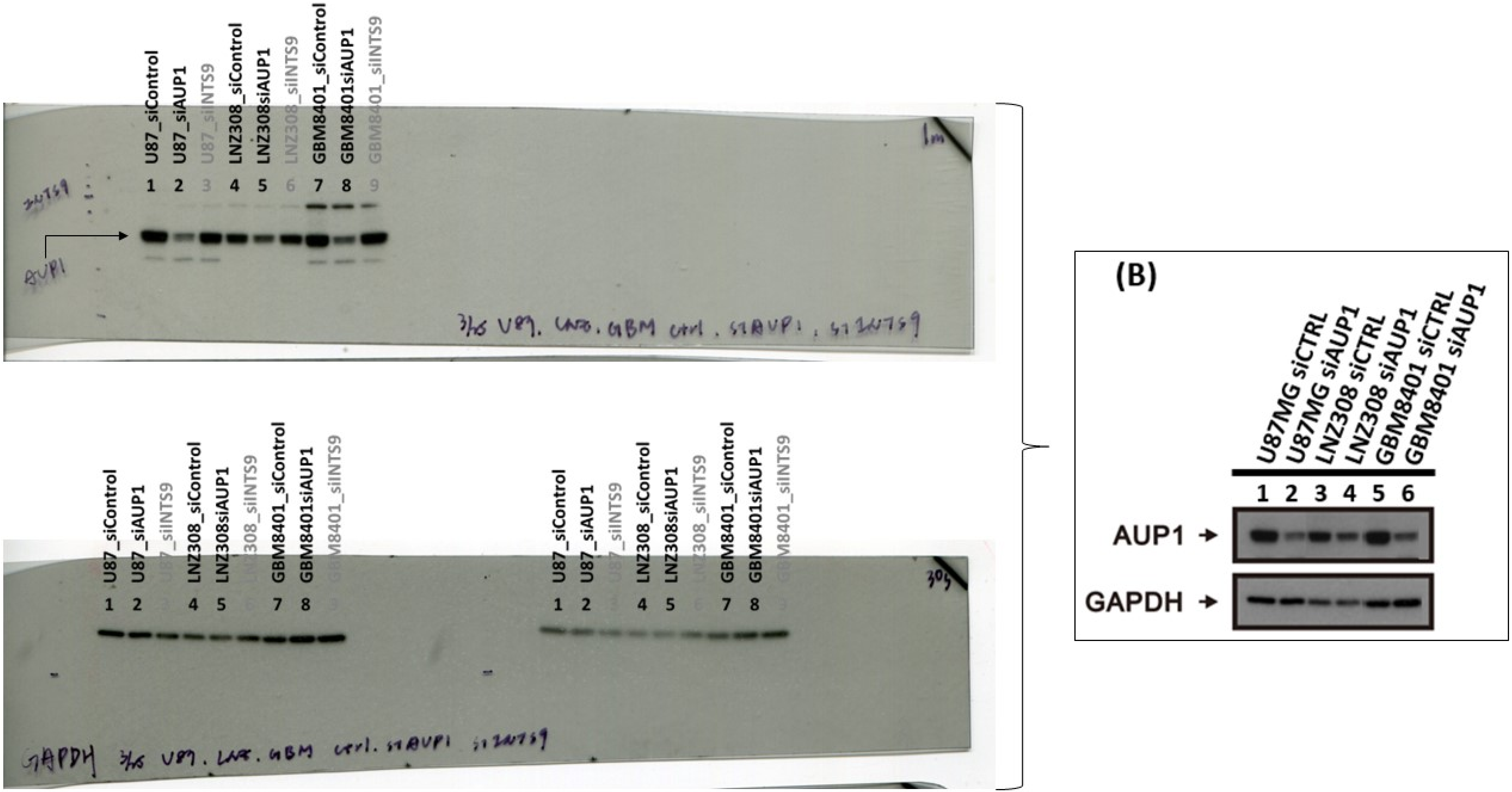

Supplementary 4 _for **Figure 5(D)**

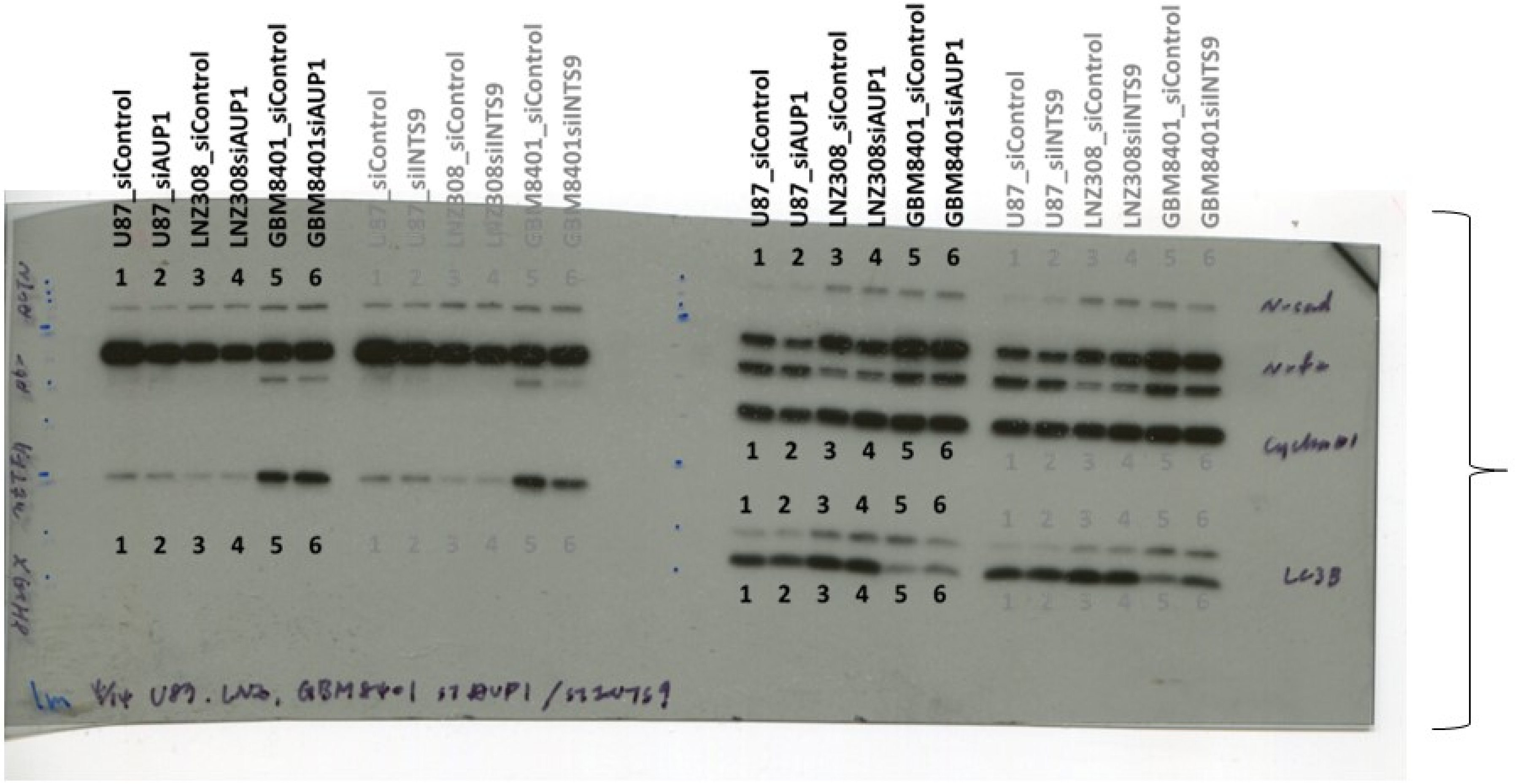

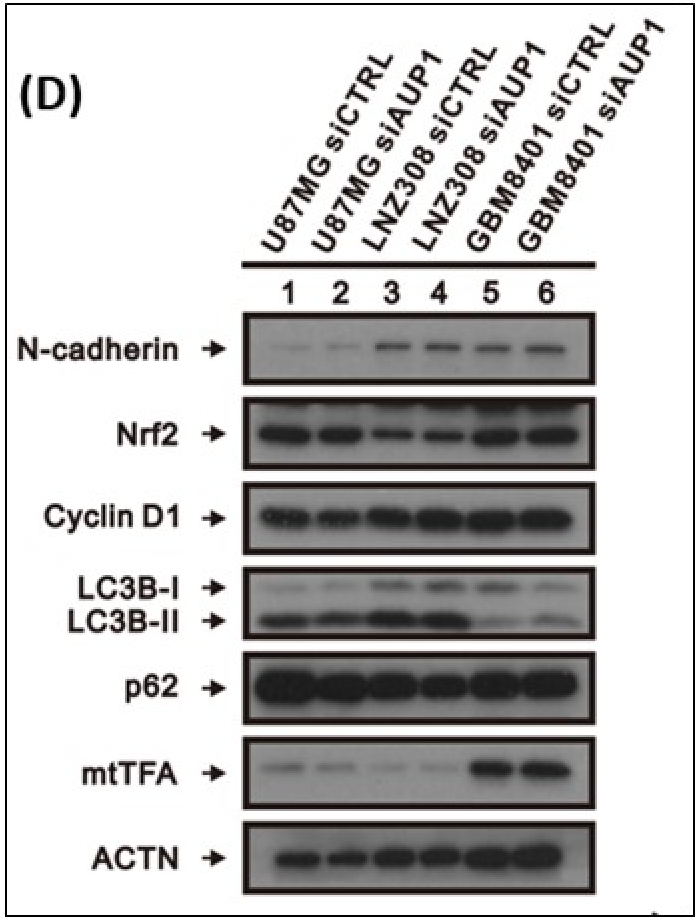

Supplementary 4 _for **Figure 5(G)**

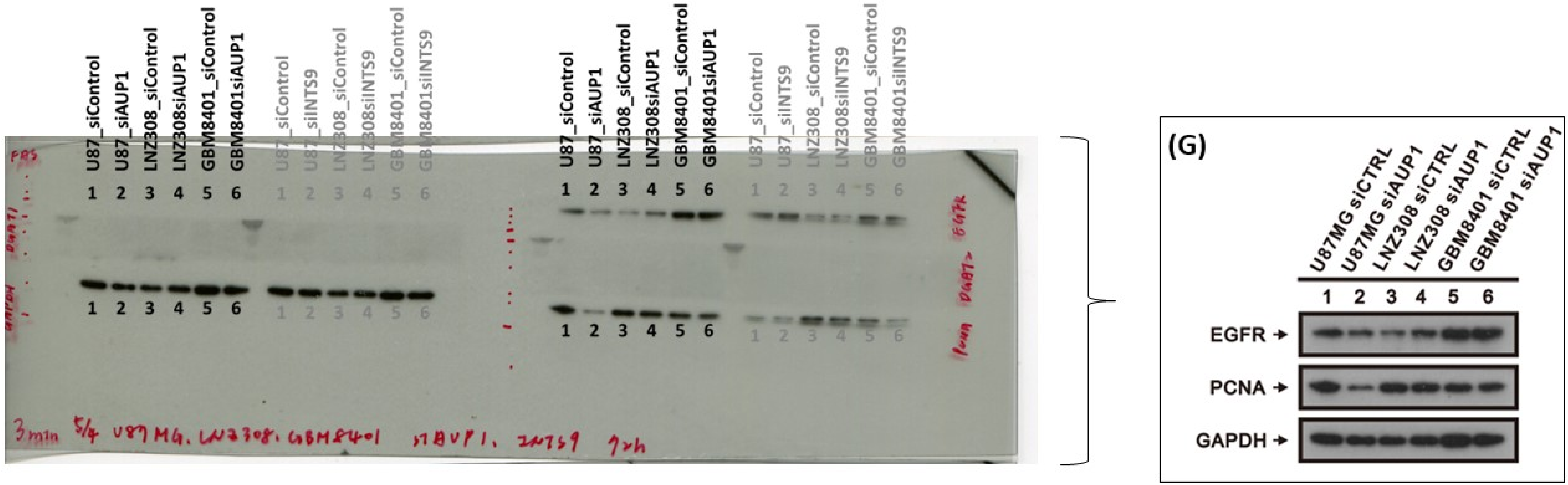

Supplement: Supplementary file 5 — Additional file 5. Original data of Western-Blot for Fig. 5. [file 12935_2023_2912_MOESM5_ESM.pdf]
